# Microglia and programmed cell death in spinal cord injury: beyond apoptosis

**DOI:** 10.3389/fcell.2025.1656732

**Published:** 2025-10-16

**Authors:** Ming Huang, Guoquan Yao, Baowen He, Xiaohu Zhou, Guoqing Liu, Wenfei Dong

**Affiliations:** Department of Anesthesiology, General Hospital of Northern Theater, Shenyang, China

**Keywords:** spinal cord injury, microglia, autophagy, ferroptosis, pyroptosis, necroptosis, neuroinflammation

## Abstract

Spinal cord injury (SCI) triggers a multifaceted cascade of cellular and molecular events that profoundly influence the extent of secondary damage. Central to this process, microglia—the innate immune cells of the central nervous system—display a range of programmed cell death pathways that have significant implications for injury outcomes. This article mainly focuses on three key programmed cell death modalities that have emerged in SCI: ferroptosis, autophagy, and pyroptosis. Ferroptosis, characterized by iron-dependent lipid peroxidation, autophagy, which can serve dual roles in cell survival and death, and pyroptosis, an inflammatory form of cell death, contribute uniquely to the progression and resolution of post-injury neuroinflammation. We examine the underlying molecular mechanisms, the regulatory networks that integrate these pathways, and how their dysregulation may exacerbate tissue damage. Moreover, potential therapeutic strategies to modulate these specific cell death processes are discussed, offering promising avenues for reducing secondary damage and enhancing recovery in patients with SCI.

## Graphical Abstract

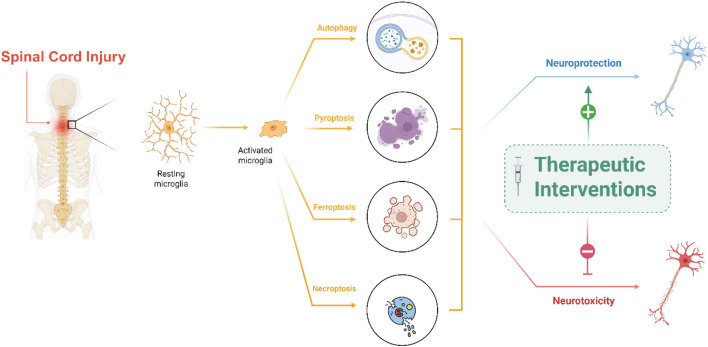

## Highlights

• Microglia undergo multiple forms of programmed cell death after spinal cord injury
• Autophagy, ferroptosis, and Pyroptosis each shape injury outcomes in distinct ways
• Disrupted autophagy links to chronic inflammation and worsened neurological damage
• Targeting death pathways can reprogram microglia toward tissue repair and recovery
• Therapeutic modulation of microglial death offers new hope for spinal cord repair

## 1 Introduction

Global incidence of spinal cord injury (SCI) has risen over the past three decades, disproportionately affecting older adults and men and producing mostly incomplete quadriplegia and high early-mortality rates (Ding et al., 2022).

Beyond the initial mechanical insult that shears axons and disrupts the vasculature, a secondary-injury cascade unfolds within minutes to months. Ionic imbalance, ischemia/reperfusion, excitotoxicity, and oxidative stress trigger waves of programmed cell death, while activated microglia, infiltrating macrophages, and cytokines drive chronic neuroinflammation that expands the lesion and inhibits axon regrowth (Kim et al., 2023; Sterner and Sterner, 2022; Begum et al., 2024).

After SCI, resident microglial cells (macrophages) are the first immune cells to react, rapidly switching to an amoeboid, phagocytic state and, together with infiltrating CCR2^+^ (C–C chemokine receptor type 2) blood-borne monocyte-derived macrophages, create an ionized calcium-binding adaptor molecule 1 (Iba1) front that both clears debris and intensifies tissue damage through Tumor Necrosis Factor-alpha (TNF-α), Interleukin-1 beta (IL-1β), and reactive oxygen species (ROS) (Timofeeva et al., 2025). Microglial cells are highly plastic. The first week is dominated by an early M1-like (iNOS^+^/CD86^+^) population. In contrast, a transient M2-like [Arg1^+^ (Arginase 1)/CD206^+^ (mannose receptor C-type 1, MRC1)] phase provides IL-10, Transforming Growth Factor-beta (TGF-β), and growth factors that aid remyelination (Wang et al., 2023). If the inflammatory response fails to resolve, chronic microglia/macrophage plateau emerges; sustained colony stimulating factor 1 (CSF-1) signaling keeps these cells within the lesion core, where they secrete extracellular matrix modulators that consolidate the glial scar and blunt axon regeneration, a barrier that can be partially lifted by delayed CSF-1 receptor inhibition (Stewart et al., 2025).

After SCI, the inflammatory milieu dysregulates homeostatic microglial processes (such as autophagy) and engages multiple regulated cell‐death pathways. Autophagy is a conserved lysosomal degradation pathway initiated by Unc-51 Like Autophagy Activating Kinase 1 (ULK1) complex activation, regulated upstream by nutrient and energy sensors such as mechanistic target of rapamycin (mTOR) and AMP-activated protein kinase (AMPK). Under stress, AMPK phosphorylates ULK1, while mTOR inhibition releases ULK1 from repression, leading to nucleation of the phagophore *via* Beclin-1 and recruitment of microtubule-associated protein 1 light chain 3 (LC3) for autophagosome formation (Liao et al., 2021).

After SCI, lysosomal dysfunction and mTOR hyperactivation impair autophagic flux in glial cells, including microglia. This inhibition is linked to the accumulation of receptor-interacting protein kinase 1 or 3 (RIPK1/RIPK3) and sensitization to necroptosis, as shown by increased RIPK1 and RIPK3 levels in injured spinal tissue and microglial cultures (Liu et al., 2018).

Pharmacological induction of autophagy—using rapamycin or resveratrol—reactivates AMPK/mTOR-Beclin-1 signaling, suppresses NF–κB–mediated cytokine production, and attenuates microglial proinflammatory activation, improving functional recovery in rodent SCI models (Liao et al., 2021). Pyroptosis is a caspase-1 (canonical) or caspase-4/5/11 (non-canonical)–mediated inflammatory cell death characterized by gasdermin D (GSDMD) pore formation, leading to cell swelling and release of IL-1β and IL-18 (Yin et al., 2022). In SCI models, damage-associated molecular patterns (DAMPs) [e.g., ATP, high-mobility group box 1 (HMGB1)] and Toll‐like receptor signals converge on NOD-like receptor family pyrin domain containing 3 (NLRP3) inflammasome assembly in microglia, recruiting apoptosis-associated speck-like protein containing a CARD (ASC) and procaspase-1 into a multiprotein complex. Activated caspase-1 cleaves pro-IL-1β/IL-18 and GSDMD, triggering pyroptotic lysis. Elevated NLRP3, ASC, caspase-1, GSDMD, IL-1β, and IL-18 expression has been documented in both SCI rat spinal cords and Lipopolysaccharide (LPS) + ATP-stimulated BV-2 microglia (Al Mamun et al., 2021; Xu et al., 2020).

Ferroptosis is driven by iron‐dependent lipid peroxidation when antioxidant defenses (notably glutathione peroxidase 4 (GPX4) and glutathione synthesis *via* system XC) fail, leading to membrane damage and cell death (Li F. et al., 2022). Recent studies reveal that iron overload and oxidative stress in the injured spinal cord promote ferroptosis in neurons and in infiltrating macrophages and resident microglia. Ferroptotic macrophages release pro‐inflammatory mediators that further prime microglia toward a pro‐inflammatory phenotype, amplifying tissue damage (Zhao et al., 2024).

Necroptosis is a caspase-independent lytic death pathway initiated when RIPK1 kinase activity is unchecked (often due to caspase-8 inhibition), leading to RIPK3 phosphorylation, mixed lineage kinase domain-like pseudokinase (MLKL) recruitment, MLKL oligomerization, and plasma membrane rupture (Hu et al., 2022). Necroptotic markers, including elevated RIPK1, RIPK3, and phosphorylated MLKL, are upregulated in spinal cord lesions and microglia isolated post‐injury. MLKL localizes to damaged endoplasmic reticulum membranes in dying microglia, linking endoplasmic reticulum stress to necroptotic execution (Fan et al., 2015).

PANoptosis is a recently described modality that integrates key components of pyroptosis, apoptosis, and necroptosis through a supramolecular complex termed the PANoptosome. It is orchestrated by innate sensors like Z-DNA binding protein 1 (ZBP1), absent in melanoma 2 (AIM2) and NLRP12, that recruit adaptors and caspases (caspase-1, 8) alongside RIPKs, simultaneously activating multiple death pathways and robust inflammatory signaling (Gao J. et al., 2024; Pandian et al., 2022). Although PANoptosis has not yet been directly demonstrated in microglia after SCI, the injury milieu, rich in DAMPs, pro‐inflammatory cytokines (TNF-α, IFN-γ), and pathogen‐associated molecular patterns, provides strong triggers for PANoptosome assembly. Microglial expression of NLRP3, AIM2, and ZBP1 increases after SCI, and caspase-8, caspase-1, RIPK1/RIPK3 are all upregulated in parallel. These observations suggest that SCI could foster conditions for microglial PANoptosis, leading to a mixed death phenotype that magnifies inflammation, disrupts tissue integrity, and impairs repair processes (Pandian et al., 2022).

## 2 Autophagy

Autophagy is a conserved cellular process in which eukaryotic cells break down and recycle their own components through the lysosomal system. It maintains cellular homeostasis by degrading macromolecules and organelles, providing energy and building blocks, especially during stress like nutrient starvation. Autophagy is vital for quality control, development, immunity, and is linked to diseases such as cancer and neurodegenerative disorders when dysregulated (Pyo et al., 2012; Hurley and Young, 2017).

**FIGURE 1:**
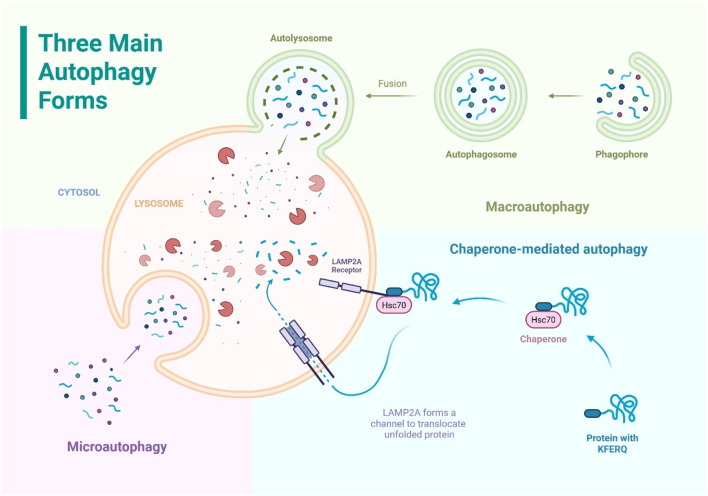
The three main forms of autophagy: macroautophagy, microautophagy, and chaperone-mediated autophagy. All three pathways deliver cytoplasmic components to the lysosome for degradation.

Eukaryotic cells exhibit three main forms of autophagy including macroautophagy, microautophagy, and chaperone-mediated autophagy, distinguished by how cargo is delivered to lysosomes (Figure 1). Each pathway contributes to cellular housekeeping, but they differ in their cargo selectivity and membrane dynamics (Pyo et al., 2012).

Macroautophagy, often referred to simply as autophagy, is a process where parts of the cytoplasm are enclosed in a double-membrane (phagophore) which expands and closes to form an autophagosome, which then fuses with a lysosome for degradation (autolysosome). It can be non-selective or selective, targeting specific damaged components like organelles or protein aggregates. This pathway is crucial for clearing large cellular debris and maintaining cell health (Yamamoto and Matsui, 2024).

**FIGURE 2:**
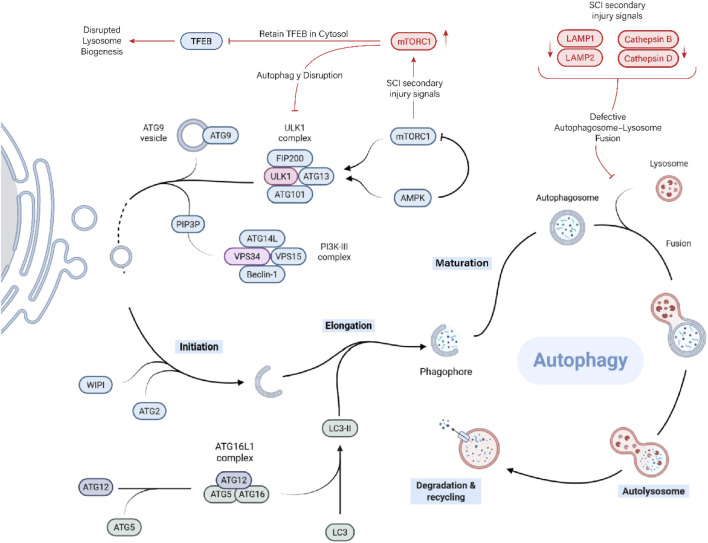
Autophagy and its disruption after SCI. Canonical autophagy—from ULK1-driven initiation and PI3K-III/Beclin-1 nucleation to LC3-II-dependent elongation, autophagosome maturation, and lysosomal degradation. After SCI, mTORC1 hyperactivation inhibits the ULK1 initiation complex and retains TFEB in the cytosol, limiting lysosome biogenesis. In parallel, lysosomal dysfunction (reduced LAMP1/2 and cathepsin B/D activity; membrane fragility) compromises autophagosome–lysosome fusion, causing a flux stall characterized by LC3-II-positive vesicle accumulation and p62/SQSTM1 buildup. The resultant defective clearance sustains microglial inflammatory signalling.

It begins with initiation, where the ULK1 complex (containing ULK1/2, autophagy-related protein 13 (ATG13), FAK family–interacting protein of 200 kDa (FIP200), and ATG101) becomes activated upon Mechanistic Target of Rapamycin (mTOR) inhibition and localizes to specific sites on the endoplasmic reticulum (Figure 2). This complex recruits ATG9 vesicles and other factors to form the phagophore assembly site (Pyo et al., 2012).

Next is nucleation, driven by the class III phosphoinositide 3-kinase (PI3K) complex (vacuolar protein sorting 34 (Vps34), Beclin-1, Vps15, and ATG14L), which produces phosphatidylinositol 3-phosphate (PI3P) at the phagophore. This attracts WD repeat domain and phosphoinositide-interacting (WIPI) proteins and ATG2 to tether endoplasmic reticulum membranes and facilitate lipid flow into the growing membrane. The elongation phase involves two ubiquitin-like conjugation systems. One conjugates ATG12 to ATG5, which then forms a complex with ATG16L1. This complex helps catalyze the lipidation of LC3, attaching it to the growing membrane as LC3-II. LC3-II supports membrane expansion and cargo loading by interacting with adaptor proteins that recognize damaged or tagged cytoplasmic components. Once the phagophore fully encircles the cargo, the membrane closes, forming a sealed autophagosome. The autophagosome is then transported to the lysosome-rich perinuclear region. Finally, in the degradation and recycling phase, the breakdown products (amino acids, fatty acids, sugars, and nucleotides) are exported back into the cytoplasm for reuse in metabolism and biosynthesis. This recycling is essential for survival during starvation and for maintaining cellular quality control. The lysosome itself can be regenerated from the autolysosome to participate in future rounds of autophagy (Yamamoto and Matsui, 2024).

Microautophagy is a process where the lysosome directly engulfs small portions of the cytosol by membrane invagination or protrusion to internalize cytosolic cargo into intraluminal vesicles within the lysosome. It can be selective or non-selective and targets structures like the nucleus, peroxisomes, or lipid droplets. Unlike macroautophagy, it does not form a separate autophagosome and often uses the Endosomal Sorting Complex Required for Transport (ESCRT) machinery for membrane remodeling (Yamamoto and Matsui, 2024).

Chaperone-Mediated Autophagy is a highly selective process that degrades specific cytosolic proteins without forming vesicles. Proteins with a KFERQ-like motif are recognized by the chaperone HSC70, which delivers them to the lysosomal receptor lysosome-associated membrane protein type 2a (LAMP2A). LAMP2A forms a channel to translocate the unfolded protein into the lysosome for degradation. Chaperone-mediated autophagy acts one protein at a time and plays a key role in protein quality control, especially during prolonged stress and in organs like the liver and kidney. This pathway is unique to higher eukaryotes (Yamamoto and Matsui, 2024; Kaushik and Cuervo, 2018).

Under nutrient-rich conditions, mechanistic target of rapamycin complex 1 (mTORC1) acts as a central inhibitor of autophagy. It senses the availability of amino acids and growth signals, and actively suppresses autophagy initiation by phosphorylating and inhibiting ULK1. As long as mTORC1 is active, ULK1 remains off, and autophagy is blocked. However, when nutrients become scarce mTORC1 activity drops, releasing this inhibition. ULK1 is then free to activate, allowing autophagy to begin. Pharmacologically, mTORC1 inhibitors like rapamycin mimic this starvation signal and potently induce autophagy (Alers et al., 2012).

In contrast to mTORC1, AMPK is a sensor of low energy (high AMP/ATP ratio) and functions as a positive regulator of autophagy. When cellular energy is low, AMPK directly activates ULK1 by phosphorylating it on distinct activating sites. At the same time, AMPK also helps shut down mTORC1, both by phosphorylating its regulatory subunits and by activating upstream inhibitors of mTORC1. Through these combined actions, AMPK not only removes the block imposed by mTORC1 but also actively promotes the initiation of autophagy. Essentially, AMPK and mTORC1 exert opposing influences on ULK1, and the balance between them determines whether autophagy is switched on or off (Alers et al., 2012).

While mTORC1 and AMPK regulate the immediate activation of autophagy, transcription factor EB (TFEB) governs the long-term capacity of the autophagy–lysosome system by controlling gene expression. However, under nutrient-rich conditions, mTORC1 phosphorylates TFEB, keeping it in the cytosol by anchoring it to 14-3-3 proteins, thus preventing it from activating its target genes (Alers et al., 2012).

Other related transcription factors, such as transcription factor binding to IGM enhancer 3 (TFE3) and microphthalmia-associated transcription factor (MITF), also contribute to this program and can compensate for TFEB when needed. Additional transcriptional regulators like Forkhead box O (FoxO) proteins, p53, and nuclear factor kappa-light-chain-enhancer of activated b cells (NF-κB) can influence autophagy in context-dependent ways, but TFEB remains the core transcriptional switch for scaling up autophagic and lysosomal function (Liu X. et al., 2021; Yu et al., 2018).

After traumatic SCI, lysosomal integrity is compromised, accumulating autophagosomes without efficient degradation, a phenomenon termed autophagy disruption. This is evidenced by increased levels of LC3-II in injured spinal tissue, indicative of stalled flux (Liu et al., 2015). Enhanced autophagic flux can clear damaged mitochondria and reduce oxidative stress, attenuating secondary injury. For instance, rapamycin‐induced autophagy improves locomotor recovery in rodent SCI models by suppressing neuronal apoptosis and inflammation (Liao et al., 2021).

Conversely, excessive or prolonged autophagy may promote autophagic cell death, particularly under severe injury conditions where energy stores are depleted (Abbaszadeh et al., 2020).

Thus, autophagy activation after SCI follows a biphasic pattern: neurons experience an early surge (within hours), and glial cells experience a secondary wave (days post-injury). In response to inflammatory cytokines, Microglia and astrocytes upregulate autophagy-related proteins, influencing lesion progression and scar formation (Zhou et al., 2017).

### 2.1 Molecular and cellular mechanisms

#### 2.1.1 NLRP3‐inflammasome and autophagy

Across SCI models, NLRP3 and autophagy are tightly coupled and bidirectionally regulated. Inhibiting NLRP3 (MCC950 or knockdown) elevates mTOR signaling, reduces autophagic flux (↓LC3-II/Beclin-1, ↑p62), limits autophagic cell death, promotes an M2-like microglial phenotype, and improves locomotion; rapamycin reverses these effects, defining a functional NLRP3–mTOR–autophagy axis *in vivo* and in BV-2 microglia (Tian et al., 2025). In contrast, targeted autophagy induction can eliminate NLRP3 itself. Cannabinoid receptor-2 (CB2) activation (JWH-133) triggers AMPK/ULK1-dependent autophagy (↑microtubule-associated protein 1 light chain 3 beta (LC3B), ↓p62), drives K48-linked ubiquitination, and routes NLRP3 to autophagosomes, yielding M2 polarization, reduced neuroinflammation/demyelination, and better recovery (Jiang et al., 2022). Mitochondria-directed strategies—urolithin A and ginkgolide B—boost putative kinase 1 (PINK1)-Parkin mitophagy, clear damaged mitochondria, lower mitochondrial ROS (mtROS), and block ASC specks, caspase-1, IL-1β, and GSDMD cleavage; 3-methyladenine (3-MA), bafilomycin a1 (BafA1), or Parkin knockdown abrogate these benefits, implicating mitophagy as a key brake on NLRP3-driven pyroptosis and pain (Chen et al., 2025; Liang et al., 2024). Zinc confers dual autophagy-centric control by suppressing long non-coding ribonucleic acid (lncRNA) XIST/upregulating miR-374a-5p to reduce NLRP3 expression and by enhancing ubiquitin-mediated autophagic degradation; autophagy blockade reinstates inflammasome activity (Zhao et al., 2022; Lin et al., 2021). MicroRNA (miRNA) regulation offers another layer of control. miR-99b-3p targets matrix metallopeptidase 13 (MMP13) to increase autophagic flux, repress NLRP3, and attenuate caspase-1/GSDMD-dependent pyroptosis, easing neuropathic pain (Gao X. et al., 2024). Photobiomodulation downregulates Toll-like receptor 2 (TLR2), normalizes autophagy markers, and dampens NLRP3–caspase-1–IL-1β signaling with motor gains (Zuo et al., 2025).

Together, these data indicate that benefit derives not from unidirectional changes in autophagy but from restoring an optimal autophagy set-point that restrains pathological inflammasome signaling without triggering autophagic cell death.

Limitations across studies include reliance on rodent models and short-term outcomes, limited cell-specific genetics and safety data, frequent use of bulk LC3-II/p62 without rigorous flux assays, and focus on single pathways despite crosstalk between mTOR/AMPK, ubiquitination, and mitophagy.

#### 2.1.2 PI3K/AKT/mTOR signalling

**FIGURE 3:**
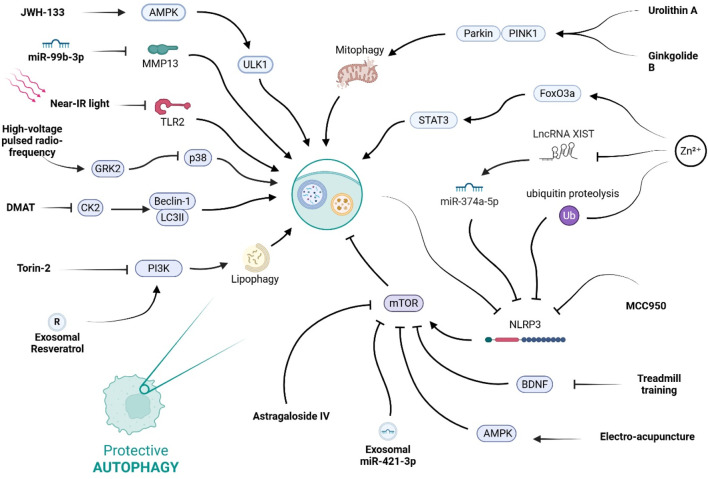
This diagram illustrates the complex regulatory network of protective autophagy. It shows how various stimuli, including natural compounds, pharmacological agents, and physical therapies, can promote this process by modulating key signaling hubs like AMPK, mTOR, and the NLRP3 inflammasome.

The PI3K/protein kinase b (AKT)/mTOR axis sits at the center of nutrient sensing and therefore at the core of autophagy regulation. In nutrient-replete cells, class-I PI3K activates AKT, which in turn activates mTORC1 (Figure 3); mTORC1 then phosphorylates the ULK1 complex, blocking autophagy initiation. Energy stress, AMPK activation, growth-factor withdrawal, or upstream inhibition of PI3K/AKT all relieve this brake and trigger autophagic flux, thereby maintaining metabolic homeostasis. Parallel modulators such as brain-derived neurotrophic factor (BDNF) (*via* tropomyosin receptor kinase B (TrkB)/AKT) and g protein-coupled receptor kinase 2 (GRK2) [which constrains p38-mitogen activated protein kinase (MAPK)] fine-tune the pathway in neural tissue, while AMPK provides an energy-status override that can suppress mTORC1 even when PI3K is active (Zhang HP. et al., 2024; Xu et al., 2023).

Several lines of evidence show that sustained PI3K/AKT/mTOR activity blocks autophagy in spinal cord macrophages and thereby aggravates secondary injury.

Lipid-engorged “foamy” macrophages that accumulate after traumatic SCI depend on PI3K activity to phagocytose myelin debris while simultaneously suppressing autophagic clearance of lipid droplets; *Torin-2* (a PI3K/mTOR inhibitor) reverses the phenotype, whereas *rapamycin* (mTORC1-specific) does not, underscoring a PI3K-dependent, mTOR-independent step in this context (Ryan CB. et al., 2024).

Bioinformatic mapping of immune subsets in injured cords identifies mTOR as a hub gene in proinflammatory M1 macrophages; increased PI3K-AKT-mTOR signalling coincides with low LC3B-II and high p62, perpetuating cytokine output (TNF-α, IL-1β) (Ju et al., 2025).

In ischemic SCI, PI3K/AKT phosphorylation falls transiently, yet further inhibition (mild hypothermia or rapamycin) is required to push autophagy high enough to curb microglial over-activation and neuroinflammation (Li J. et al., 2022).

*Astragaloside IV* dampens mTORC1, restores neuronal autophagic flux, and switches microglia toward an M2 phenotype, together improving functional recovery (Lin et al., 2020).

Inhibiting casein-kinase-2 with *Dimethylamino-4,5,6,7-tetrabromo-1H-benzimidazole* elevates Beclin-1/LC3II, lowers p62, and forces M1 to M2 microglial repolarization; blocking autophagy with *3-MA* abolishes these effects, proving autophagy is the mechanistic bridge (Liu et al., 2023).

Microglia-derived exosomes loaded with *resveratrol* enter the spinal cord, raise LC3B and Beclin-1, and repress apoptosis *via* PI3K activation; *3-MA* negates the benefit, confirming pathway dependence (Fan et al., 2020).

Small extracellular vesicles (sEVs) from M2 bone-marrow macrophages deliver miR-421-3p to neurons, directly targeting mTOR and thereby restoring autophagic flux and reducing cell death (Wang et al., 2020).

Voluntary treadmill running suppresses spinal BDNF, releases the AKT/mTOR brake, expands autophagic flux in microglia, favors M2 polarization, and lessens neuropathic pain (Bai et al., 2022).

High-voltage pulsed radiofrequency (HVPRF) applied to dorsal root ganglia reinstates GRK2, inhibits p38, restores autophagic flux in microglia, and alleviates both pain and depressive-like behavior; GRK2 knock-down reverses these gains (Xu X. et al., 2024).

Separate HVPRF work shows an autophagy-dependent drop in TNF-α and rise in IL-10 within dorsal horn microglia, correlating with structural nerve repair (Chen et al., 2024).

Electroacupuncture (EA) activates AMPK, suppresses mTOR, clears p62 in dorsal-root-ganglion macrophages, and produces potent analgesia (Xu et al., 2022).

Zinc drives Signal Transducer and Activator of Transcription 3 (STAT3)/Forkhead box O3a (FoxO3a)-dependent mitophagy, restores mitochondrial ATP output, and reduces ROS in microglia, indirectly easing secondary damage (Cui et al., 2025).

Collectively, these studies shows that PI3K/AKT/mTOR pathway operates as a molecular regulator on autophagy after SCI. Hyper-activation of PI3K or mTOR sustains lipid accumulation, inflammatory cytokine release, and neuropathic pain, whereas targeted inhibition (pharmacological, genetic, or physical) re-engages autophagic clearance and tilts immune cells toward a reparative M2 profile. Importantly, several interventions (Torin-2, AMPK activation, BDNF suppression, GRK2 restoration) achieve benefits that *rapamycin* alone does not, highlighting PI3K- or upstream-specific checkpoints and cross-talk with energy-sensing modules.

However, most data come from rodent models. Human verification is urgently needed. The temporal window for safe mTOR inhibition *versus* essential anabolic repair remains undefined. Few studies address long-term outcomes beyond acute or sub-acute phases. The differential roles of resident microglia *versus* infiltrating macrophages are not consistently dissected, leaving cell-type specificity uncertain.

Comparative studies using conditional knock-outs or cell-specific drug delivery could clarify lineage-specific requirements. Combining PI3K/mTOR modulation with mitophagy boosters or metabolic support may further refine therapeutic windows. Finally, dose-controlled clinical trials of pathway modulators such as metformin, alongside biomarker-guided imaging of autophagic flux, are warranted to translate these promising pre-clinical findings into neuro-restorative strategies for patients with SCI.

#### 2.1.3 Autophagy as a multidimensional regulator of inflammatory, hypoxic, metabolic, epigenetic, cell cycle, and vascular signaling

Autophagy functions as a hub that tunes microglial and vascular responses after SCI. On the inflammatory axis, excess lipid-peroxidation product 4-hydroxy-2-nonenal (4-HNE) (in aldose-reductase deficiency) covalently modifies IKK—especially IKKα—diverting it to p62-mediated autophagolysosomal degradation, thereby suppressing canonical NF-κB signalling and limiting secondary damage; at sub-toxic levels, 4-HNE transiently activates NF-κB (Han et al., 2022). Restoring lysosomal competence with progranulin (↑LAMP1 (lysosome-associated membrane protein 1), cathepsin D) re-opens autophagic flux (↑Beclin-1, ↑LC3-II/I, ↓p62), promotes an M2 shift, reduces NF-κB–dependent cytokines, and elevates IL-10 (Shi et al., 2022).

Hypoxia/ischemia imposes a biphasic program: brief hypoxia-inducible factor-1α (HIF-1α) stabilization upregulates Beclin-1/LC3 to curb early TNF-α/IL-1β, whereas sustained HIF-1α overdrives flux and precipitates autophagy-dependent death; late HIF-1α dampening rescues viability but re-elevates cytokines (Wang et al., 2017). After ischemia-reperfusion (I/R), an <24 h “early-autophagy” window is protective, while by 72 h persistent Beclin-1/LC3-II with reduced Bcl-2 signals autophagic cell death (Fang et al., 2016). Super-resolution structured-illumination microscopy adds spatial detail to this temporal story. lysosomes shift from subplasmalemmal clusters at 48 h to a perinuclear configuration by day 7, aligning trafficking with a transition from phagocytosis to *bona fide* autophagy (Fumagalli et al., 2019). HIF-1α-driven estrogen-related receptor-α (ERRα) further tempers p38-MAPK/NF-κB, narrows Beclin-1/LC3 windows while restoring p62, and promotes Fibronectin type III domain–containing protein 5 (FNDC5)-mediated BDNF release, coupling metabolic control to anti-inflammatory signalling and survival (Deng et al., 2022).

Myelin-derived lipids push microglia into a lipid-droplet–accumulating state with sluggish phagocytosis; miR-223 silences cathepsin B to enhance lipophagy, accelerate droplet clearance, and suppress IL-1β (Ma et al., 2024). Conversely, CD36 upregulation blocks AMPK and TFEB, stalling autophagosome biogenesis and lysosome formation; CD36 inhibition restores lipophagy, limits pyroptosis, and improves locomotion (Wang BN. et al., 2025). Systemically, M1-skewed macrophage/microglia deliver nitric oxide (NO) to Leydig cells, shutting down AMPK/ULK1-S555 and activating mTOR/ULK1-S757, freezing lipophagy and causing hypogonadism; Scavenging NO (cPTIO) or ULK1 agonism reverses this (Zhuang et al., 2024).

Epigenetic control shapes these programs: single-cell maps place autophagy genes (cathepsin D, CD68, CD81, Tyrobp) in phagocytic MG2 microglia, while histone deacetylase 3 (HDAC3) in proliferative MG4 coordinates autophagy-immune networks; HDAC3 inhibition re-routes trajectories and dampens these transcripts, implicating chromatin-level regulation of autophagy and inflammation (Wahane et al., 2021). In microvascular endothelial cells, injury-induced histone deacetylase 6 (HDAC6) elevation impairs autophagosome–lysosome fusion; Tubastatin-A restores flux, enhances myelin clearance, and reduces inflammation (Wu et al., 2024a).

Cell-cycle control intersects *via* cyclin-dependent kinase 1 (CDK1). Elevated in chronic SCI, it drives autophagy and microglial activation; knockdown lowers LC3/Beclin-1, curbs apoptosis, and is neuroprotective (Nie et al., 2022).

Vascular-immune crosstalk also hinges on autophagy: M1 microglia upregulate lymphatic vessel endothelial hyaluronan receptor-1 (LYVE-1)/podoplanin and secrete vascular endothelial growth factor-C (VEGF-C), which feeds back *via* vascular endothelial growth factor receptor-3 to reinforce M1 polarization while suppressing autophagy (Xian et al., 2025); endothelial autophagy clears opsonized myelin *via* ATG5-dependent machinery but triggers IL-6/monocyte chemoattractant protein-1 (MCP-1)/VEGF release, TGF-β1-driven endothelial-to-mesenchymal transition, and scarring (Zhou et al., 2019); and iron overload (IRP1↑ (Iron Regulatory Protein 1), transferrin-receptor-1–mediated import, ferritin↓) activates NF-κB/autophagy/cytokine cascades that injure neurons and drive pain, mitigated by chelation, NOS blockade, or microglial silencing (Meng et al., 2017).

Taken together, therapeutic benefit derives not from uniformly raising or lowering autophagy, but from tuning its timing, location, and metabolic context to restrain NF-κB–driven inflammation, avoid autophagic death, and coordinate vascular remodeling after SCI.

### 2.2 Pathological consequences

Autophagy dysfunction after SCI is a unifying driver of secondary degeneration, systemic comorbidities, and maladaptive pain states. Evidence across models underscores how disrupted flux—whether too little, too much, or mistimed—amplifies inflammation, destabilizes metabolism, and distorts neural signaling.

Immediately post-injury, microglia in the dorsal cord show an early, transient blockade of autophagy. LC3-II and p62 accumulate, cathepsin-D drops, and lysosomes fail to degrade cargo. In motor neurons, this couples to ER-stress markers (GRP78, CHOP, ATF4) and caspase-12/3 apoptotic cascades, which later subside only once cathepsin-D recovers—defining a narrow therapeutic window (Liu et al., 2015). Genetic impairment reinforces this; Beclin-1 haplo-insufficiency worsens inflammasome activation, cyclic GMP-AMP synthase (cGAS)/stimulator of interferon genes (STING) signalling, cytokine output, and motor decline, whereas trehalose restores flux, dampens these responses, and improves recovery (Li Y. et al., 2022). Downstream, microglia amplify secondary tissue loss *via* TNF-α, NO, and glutamate, killing oligodendrocytes and activating astrocytes to produce proteoglycan-rich scars. Suppressing microglial activation with the inhibitory peptide TKP reduces apoptosis, astrogliosis, scar formation, and preserves function (Emmetsberger and Tsirka, 2012).

Age exacerbates this vulnerability. Aged mice sustain broader cortical and hippocampal damage after SCI, with heightened motor and cognitive deficits. Their microglia exhibit autophagic vesicle build-up (LC3, p62, ATG7, LAMP1) consistent with lysosomal stalling, which drives excessive phagocytosis of synapses and neurons, thereby worsening neurodegeneration (Lei et al., 2024). Peripheral consequences also emerge. In rats, SCI-provoked M1 macrophages infiltrate testes, releasing NO that disrupts Leydig-cell lipophagy through AMPK/ULK1 inhibition and mTOR/ULK1-S757 activation. The resulting lipid accumulation blocks steroidogenesis, causing testosterone loss and infertility. NO scavenging or ULK1 agonists restore lipophagy and reproductive function (Zhuang et al., 2024).

Microglial autophagy likewise regulates nociception. After spinal nerve ligation, Toll-like receptor 3 (TLR3) upregulation enhances LC3-II flux and p62 clearance, boosting cytokines and pain hypersensitivity; silencing TLR3 or autophagy blockade reverses this (Chen and Lu, 2017). By contrast, miR-15a replenishment represses AKT3 (one of the protein kinase B isoforms), reactivates autophagy, suppresses cytokine release, and normalizes thresholds in chronic constriction injury (Cai et al., 2020). Resolvin D2 extends this “pro-autophagic analgesia”. it lowers miR-155, restores PTEN, reopens autophagy, and repolarizes microglia toward M2, reducing pain and improving locomotor outcomes (Yang L. et al., 2023). Yet autophagy can also be maladaptive. In morphine tolerance, ROS/NF-κB-induced autophagy and cathepsin-B activation in dorsal-horn microglia erode GABAergic tone, enhancing glutamatergic excitation and blunting opioid analgesia. Inhibiting autophagy or cathepsin-B preserves morphine efficacy (Hayashi et al., 2014).

While most studies emphasize acute and sub-acute SCI pathology, the chronic phase (months to years’ post-injury) is marked by persistent autophagy defects, maladaptive microglial activation, and glial scar consolidation. Long-term impairment of autophagic flux in microglia and astrocytes has been linked to sustained extracellular matrix deposition, chondroitin-sulfate proteoglycan accumulation, and glial scar thickening. This structural barrier not only isolates the lesion but also inhibits axonal sprouting and functional regeneration.

In parallel, defective autophagy intersects with other death pathways to exacerbate neuropathic pain. Persistent inflammasome activity, iron dys-homeostasis, and maladaptive microglial phenotypes maintain chronic pain circuits, while impaired axonal regrowth reinforces sensory dysfunction. Importantly, prolonged microglia–astrocyte interactions extend beyond the lesion site, reshaping spinal networks and contributing to both motor disability and sensory hypersensitivity.

Therapeutic strategies that sustain or restore autophagic flux in the chronic phase—such as prolonged mTOR inhibition, TFEB activation, or epigenetic modulation—have shown promise in animal models for reducing neuropathic pain and partially lifting the inhibitory glial barrier. However, translational evidence in humans is still lacking, and future work must address whether targeting long-term autophagy defects can simultaneously mitigate pain and promote regeneration.

Together these findings show microglial autophagy as a double-edged regulator across pathological dimensions. Impaired flux fuels secondary degeneration, inflammatory scarring, and senescent neurodegeneration; blocked lipophagy links SCI to systemic endocrine failure; over- or under-activated pathways shape pain chronically, either amplifying hypersensitivity or undermining opioid therapy. Thus, the key therapeutic challenge is not simply to drive or arrest autophagy but to recalibrate its timing, intensity, and cellular context to mitigate pathology and promote recovery.

### 2.3 Therapeutic and experimental interventions

#### 2.3.1 Small-molecule drugs (synthetic or repurposed)

A growing pharmacological strategy for SCI focuses on restoring the microglial autophagy–inflammation balance. Drugs that either inhibit the canonical mTOR brake on autophagy, activate upstream energy sensors, or modulate chemokine or cell-cycle signalling have all shown the capacity to tilt microglia away from a chronically proinflammatory state and thereby limit secondary neurodegeneration.

Across two independent rat SCI studies, systemic or intrathecal rapamycin consistently suppressed mTORC1 (↓p-p70S6K), lowered the autophagy cargo adaptor p62/sequestosome-1, and increased Beclin-1 and LC3-II accumulation, indicating restored autophagic flux. Concomitantly, microglia/macrophage infiltration (CD11b^+^/CD14^+^) and TNF-α secretion fell, while neuronal survival and axon regrowth improved. A modest rise in phosphorylated AKT further supported pro-survival signalling. Histology confirmed abundant autophagic vacuoles within phagocytic cells and reduced cavitation, identifying rapamycin as a dual autophagy enhancer and inflammation dampener in the injured cord (Firat et al., 2021; Goldshmit et al., 2015).

Metformin drove a phenotypic switch from M1 to M2 microglia, boosted myelin-debris phagocytosis and preserved white matter. Mechanistically, AMPK activation and secondary mTOR inhibition re-established autophagosome–lysosome fusion; blocking autophagy with 3-MA abolished these benefits, underscoring autophagy as the primary driver of metformin’s neuroprotection (Wu et al., 2022).

Trehalose prevented the accumulation of lipid-laden “foamy” macrophages whose defective autophagy perpetuates scarring. By up-regulating TFEB, it coordinately induced autophagy- and lysosome-related genes, restored lipid clearance, curtailed proinflammatory cytokines, reduced fibrotic borders and improved motor scores. Chloroquine negated these gains, confirming TFEB-dependent autophagy as the key target (Ma et al., 2025).

The C-C chemokine receptor type 1 antagonist, BX471, dampened microglial activation and TNF-α/IL-1β release while simultaneously increasing Beclin-1 and LC3-II and lowering p70S6K (70-kDa ribosomal protein S6 kinase) and p62, thereby re-establishing autophagic flux and limiting neuronal apoptosis (Hasan et al., 2025).

In a chronic SCI model, heightened CDK1 expression paralleled excessive autophagy and microgliosis. CDK1 shRNA curtailed LC3/Beclin-1 accumulation and Iba1 levels, suggesting that aberrant CDK1 activity sustains both autophagic stress and inflammatory activation long after the initial insult (Nie et al., 2022).

Collectively, these studies highlight autophagy restoration as a unifying mechanism by which diverse small molecules blunt microglia-driven secondary injury. Rapamycin and metformin target the canonical AMPK/mTOR axis and therefore possess predictable metabolic side-effects but robust efficacy across time points. Trehalose and BX471 reveal alternative entry points, lysosomal biogenesis and chemokine signalling, broadening the therapeutic palette and potentially reducing systemic immunosuppression. CDK1 inhibition, while mechanistically intriguing, still lacks pharmacological tractability and a clear safety profile.

Major limitations remain. All data are pre-clinical, with short follow-up windows and variable dosing paradigms. Most studies assess single-agent therapy, despite the multifactorial nature of SCI. Autophagy read-outs are largely surrogate (LC3 puncta, p62), not dynamic flux measurements *in vivo*. Future work should standardize injury models, incorporate sex and age variables, employ real-time autophagy reporters, and test rational drug combinations to determine whether convergent autophagy modulation translates into durable neurological recovery.

#### 2.3.2 Natural phytochemicals and nutraceuticals

A growing body of work shows that plant-derived small molecules can fine-tune microglial autophagy after SCI, thereby curbing neuroinflammation, limiting secondary neuronal loss and, ultimately, improving functional outcome. Although their chemical scaffolds differ, most of these agents converge on two interconnected nodes highlighting common points of therapeutic leverage. Astragaloside IV, fisetin and salidroside all enhance autophagic flux by dampening mTORC1 activity, yet each does so through a slightly different upstream switch.

Astragaloside IV lowers p-mTOR and p-p70S6K without altering p-AKT, implicating AMPK as the likely brake on mTORC1. The net result is M2-skewed microglia, reduced cytokine release and concomitant protection of neurons from apoptosis, translating into better locomotor scores after SCI (Lin et al., 2020).

Fisetin directly activates AMPK, which in turn represses mTOR, restores autophagic flux and suppresses TNF-α/IL-6 output from M1-polarized microglia. This dual action relieves neuronal apoptotic pressure and accelerates behavioral recovery (Liu Y. et al., 2024).

Salidroside also recruits AMPK to block mTORC1, tipping microglia toward an M2 phenotype while curbing iNOS and cyclooxygenase-2 (COX-2) expression. Enhanced autophagy appears to be the linchpin linking salidroside’s anti-inflammatory and neuroprotective effects (Wang et al., 2018).

Clearing dysfunctional mitochondria is another recurring strategy. Ginkgolide B activates the PINK1–Parkin pathway, promoting mitophagy in microglia, lowering mtROS, preventing NLRP3 inflammasome activation and, consequently, easing neuropathic pain. Genetic or pharmacological blockade of Parkin abolishes its benefit, underscoring mitophagy as the critical driver (Liang et al., 2024).

Urolithin A, a gut-derived ellagitannin metabolite, similarly boosts PINK1-Parkin-dependent mitophagy, thereby dampening ROS-mediated NLRP3 priming, Caspase-1 cleavage and GSDMD-driven pyroptosis. The protection disappears when autophagy is blocked by 3-MA, again pinpointing mitophagy as the therapeutic nexus (Chen et al., 2025).

Some nutraceuticals modulate autophagy indirectly by correcting upstream inflammatory circuits. Resolvin D2 interrupts NF-κB-induced miR-155 upregulation, restores PTEN, inhibits AKT/mTOR and re-opens autophagy flux. With the block removed, microglia revert to an M2-like state and neuropathic pain is eased in SCI models (Yang L. et al., 2023).

Leveraging microglia themselves as drug carriers can amplify phytochemical efficacy. Microglia-derived exosomes loaded with resveratrol cross the injured cord, stimulate neuronal autophagy through PI3K signalling and suppress apoptosis, producing marked motor recovery. Inhibition of PI3K or autophagy neutralizes these gains, confirming pathway specificity (Fan et al., 2020).

Collectively, these studies reinforce the centrality of autophagy, especially AMPK–mTOR regulation and PINK1-Parkin-mediated mitophagy, in dictating microglial phenotype and downstream neuro-inflammatory tone after SCI. The convergence of chemically diverse phytochemicals on a limited set of intracellular hubs strengthens the rationale for targeting autophagy but also raises several caveats. First, most data derive from single-agent, short-term rodent experiments; the durability of benefit and potential off-target metabolic effects in larger animals or humans remain unknown. Second, because nearly every compound alters the AMPK/mTOR axis, disentangling autophagy-specific actions from broader metabolic consequences (e.g., altered glycolysis or lipid utilization) will require more nuanced readouts. Third, the favorable results with exosome-packaged resveratrol highlight the importance of delivery and bioavailability—an under-explored variable for many phytochemicals that suffer from poor solubility or rapid clearance. Finally, head-to-head comparisons and combination trials are lacking; such work is essential to determine whether these agents offer additive or redundant benefits. In summary, natural products constitute a promising, mechanistically coherent toolkit for re-programming microglial autophagy after SCI, but their translation will hinge on rigorous pharmacokinetic optimisation, long-term safety studies and careful pathway deconvolution.

#### 2.3.3 Metal ions and trace-element therapy

Microglia are among the first responders to SCI, yet they rapidly succumb to mitochondrial fragmentation, oxidative stress, and energy failure. Restoring mitochondrial quality control has therefore become a key therapeutic goal, and zinc has emerged as the most extensively studied trace element in this context (Cui et al., 2025).

In a recent mouse SCI model, systemic zinc supplementation reinstated mitophagy and preserved mitochondrial membrane potential by activating a STAT3/FoxO3a/Superoxide Dismutase 2 (SOD2) axis. Zinc curtailed FoxO3a phosphorylation, allowing its nuclear import and the transcription of antioxidant (SOD2) and fusion/biogenesis genes (peroxisome proliferator-activated receptor gamma coactivator 1-alpha (PGC-1α), optic atrophy protein 1 (OPA1), Mitofusin 2), while simultaneously suppressing the Fission Protein Dynamin-related Protein 1. The net effect was reduced mtROS, improved ATP production, and better locomotor recovery (Cui et al., 2025).

A complementary study showed that zinc preferentially expands a vascular endothelial growth factor A (VEGF-A)-secreting microglial subcluster (MG4). The released VEGF-A engages PI3K/AKT/Bcl-2 signaling in both microglia and endothelial cells, fostering endothelial proliferation, attenuating microglial apoptosis, and alleviating local hypoxia. Consequently, vascular regeneration and axonal sparing are enhanced, highlighting a dual mitochondrial-vascular mechanism by which zinc remodels the post-traumatic niche (Deng et al., 2023).

Beyond dietary Zinc, methylprednisolone—still the only FDA-approved acute anti-inflammatory for SCI—can harness endogenous zinc to induce Beclin-1–dependent autophagic death of over-activated microglia. The glucocorticoid upregulates the zinc importer Zrt/Irt-like Protein 8 (ZIP8), raises the labile‐zinc pool, suppresses NF-κB, and triggers a protective wave of autophagy. Chelation with Tetrakis (2-pyridulmethy-1) Ethylenediamine (TPEN) abolishes these benefits, underscoring zinc’s central role in the drug’s neuroprotective profile (Li et al., 2016).

Collectively, these studies converge on zinc as a pleiotropic modulator that couples mitochondrial repair, angiogenesis, and immunomodulation after SCI. By acting both as a micronutrient and as a mobilizable second messenger, zinc targets canonical (STAT3/FoxO3a) and non-canonical (PI3K/AKT/Bcl-2, Beclin-1) autophagy checkpoints, while simultaneously shaping the vascular microenvironment. However, several gaps remain. First, the dose-response window between beneficial mitophagy and detrimental microglial loss is still ill-defined. Second, most evidence derives from young male rodents; age-, sex-, and comorbidity-dependent zinc homeostasis warrants systematic study. Finally, cross-talk with other trace elements (e.g., copper, iron, manganese) and with zinc transporters in peripheral immune cells is largely unexplored. Addressing these issues will be critical before “zinc-centric” therapies can be translated into precision medicine for human SCI.

#### 2.3.4 Stem-cell, stromal-cell and extracellular-vesicle approaches

Hypoxia-preconditioned adipose-derived Mesenchymal Stem Cell (MSC) exosomes deliver circ-Astn1 into injured spinal cords, where the Circular RNA sequesters miR-138-5p and thereby de-represses ATG7. The Circular RNA ASTN1/miR-138-5p/ATG7 axis re-engages autophagy in microglia, blunts inflammation-linked apoptosis, and translates into superior neurological recovery; genetic disruption of any node in the axis abolishes the benefit (Shao et al., 2025).

Bone-marrow MSC exosomes rich in miR-21a-5p silence the E3 ubiquitin ligase PELI1, a brake on autophagy. Restored autophagic flux suppresses NLRP3 inflammasome activation, limits microglial pyroptosis, and accelerates motor recovery after SCI (Gu et al., 2024).

MSC-derived EVs carrying miR-99b-3p mitigate neuropathic pain by dampening PI3K/AKT/mTOR signalling—an autophagy-inhibitory axis. Upregulation of LC3-II and Beclin-1, coupled with p62 clearance, curbs LPS-driven microglial activation and inflammation *in vivo*, easing pain behaviors (Gao et al., 2023).

Olfactory-mucosa MSCs foster a switch from proinflammatory M1 to reparative M2 microglia through paracrine, exosome-mediated cues. Autophagy appears permissive for this shift, as reflected by reciprocal NOS2/ARG1 expression and enhanced axonal regrowth in SCI models (Wang X. et al., 2024).

Peripheral macrophage-derived exosomes likewise promote M2 polarization by shutting down PI3K/AKT/mTOR in recipient microglia, heightening autophagy, dampening cytokine output, and preserving neurons—together improving functional outcome (Zhang et al., 2021).

sEVs from alternatively activated (M2) bone-marrow macrophages deliver miR-421-3p to neurons; by directly targeting mTOR, the miRNA boosts autophagic flux and reduces apoptosis, translating into substantial locomotor improvement post-SCI (Wang et al., 2020).

Neural stem-cell (NSC)-derived sEVs provide the scaffold protein 14-3-3τ, which binds Beclin-1 to nucleate autophagosomes. Heightened autophagy lowers neuronal apoptosis and glial inflammation; over-expression or knock-down of 14-3-3τ, respectively, magnifies or blunts these gains (Rong et al., 2019a).

A complementary NSC-sEV study showed that broad autophagy activation—evidenced by LC3B and Beclin-1 upregulation with p62 loss—suppresses NO release and proinflammatory cytokines in microglia; the protective effect disappears when autophagy is blocked with 3-MA (Rong et al., 2019b).

Finally, microglia can be coaxed to release resveratrol-loaded exosomes that cross the blood-spinal cord barrier (BSCB). The phytochemical cargo activates PI3K-dependent autophagy in spinal neurons, curtails apoptosis, and markedly improves hind-limb function; PI3K inhibition negates these effects (Fan et al., 2020).

Across diverse cell sources—mesenchymal, neural, and immune—extracellular vesicles converge on a single therapeutic motif which is reinstating autophagy to reprogram neuroinflammation and promote tissue preservation after SCI. Most studies identify a miRNA or protein “switch” (miR-138-5p, miR-21a-5p, miR-99b-3p, miR-421-3p, 14-3-3τ, resveratrol) that ultimately suppresses the PI3K/AKT/mTOR axis or liberates core autophagy genes (ATG7, Beclin-1). Functional gains are robust in rodent models, yet several gaps remain. Direct head-to-head comparisons of EVs sources and loading strategies are lacking. Dosing, biodistribution, and manufacturing reproducibility are seldom addressed. long-term safety and off-target autophagy induction in non-neural tissues remain unknown. Future work should standardize EVs production, dissect combinatorial cargos, and advance toward large-animal and, ultimately, clinical testing to clarify whether autophagy-centric EVs therapy can fulfil its translational promise in human SCI.

#### 2.3.5 Gene, miRNA and protein delivery

Targeted delivery of genes, non-coding RNAs, and trophic proteins is emerging as a precise way to recalibrate microglial autophagy-inflammation crosstalk after SCI. By fine-tuning key signalling nodes these approaches dampen secondary neuroinflammation and neuropathic pain while sparing (or even rescuing) neighboring neurons.

miR-15a restores autophagy by silencing AKT3. In the chronic constriction injury (CCI) model, miR-15a is markedly downregulated, a change that coincides with heightened cytokine release and stalled autophagic flux in dorsal-horn microglia. Intrathecal delivery of a miR-15a agomir reverses this deficit. AKT3 expression falls, Beclin-1 and LC3-II rise, p62 declines, and pain behaviors improve. These data place the miR-15a/AKT3 axis upstream of microglial autophagy and identify it as a tractable target for neuropathic pain (Cai et al., 2020).

miR-99b-3p suppresses NLRP3-driven pyroptosis *via* MMP13 inhibition. CCI also triggers NLRP3 inflammasome activation, caspase-1 cleavage, and IL-1β release. Over-expressing miR-99b-3p directly represses MMP13, thereby enhancing autophagic flux (↑LC3-II, ↓p62). Restored autophagy, in turn, dampens inflammasome priming and reduces neuropathic pain behaviors, highlighting a feed-forward loop in which miR-99b-3p curtails pyroptosis by promoting autophagy (Gao X. et al., 2024).

Glial cell line–derived neurotrophic factor (GDNF) gene therapy blocks p38-MAPK/Protein Kinase C (PKC) signalling to quell microglial activation. GDNF plasmid delivery mitigates CCI-induced microgliosis, down-regulating Beclin-1, IL-1β, IL-6, and MMP-9. Mechanistically, GDNF suppresses p38-MAPK and PKC pathways, leading to reduced autophagy initiation in over-activated microglia, lower neuronal apoptosis, and attenuated mechanical allodynia (Chou et al., 2014).

Collectively, these studies converge on a unifying theme. Over-activated microglia in SCI sit at a tipping point where too little autophagy sustains inflammasome signalling and chronic pain, whereas carefully re-engaging autophagic flux normalizes the inflammatory milieu. Both miRNA replacement (miR-15a, miR-99b-3p) and trophic-factor gene delivery (GDNF gene) succeed by hitting distinct molecular switches (AKT3, MMP13, p38/PKC) yet ultimately funnel their effects through the same autophagy-centered checkpoint. While encouraging, important caveats remain—small sample sizes, single-sex cohorts, and limited long-term read-outs leave efficacy and safety unanswered for chronic phases of SCI. Future work should compare combinatorial or sequential delivery of miRNAs and neurotrophic genes, test off-target effects in larger animal models, and couple behavioral improvements to unbiased single-cell ‘omics to confirm microglia-specific reprogramming.

#### 2.3.6 Physical/neuromodulatory strategies

Physical and neuromodulatory interventions use non-pharmacological energy to re-engage the autophagy pathway in microglia or peripheral macrophages, thereby dampening neuroinflammation, limiting secondary tissue loss, and reducing neuropathic pain after SCI. A unifying theme across the studies below is that restoring autophagic flux realigns the balance between pro- and anti-inflammatory signaling.

In a spared-nerve-injury model, 85 V HVPRF restored autophagic flux in dorsal-horn microglia, lowered TNF-α, raised IL-10, repaired ganglion ultrastructure, and produced robust analgesia. The data point to a TNF-α/IL-10 neuro-immune shift driven by enhanced formation and clearance of autophagosomes and autolysosomes (Chen et al., 2024).

Using the same modality, Xu et al. showed that nerve injury suppresses GRK2, leading to p38 MAPK over-activation and stalled autophagic flux. HVPRF reinstated GRK2, curtailed p-p38, reinvigorated autophagy, and simultaneously relieved mechanical allodynia and depression-like behavior; GRK2 knock-down abrogated all benefits, identifying GRK2 as an obligatory switch in the autophagy-pain loop (Xu X. et al., 2024).

Zou et al. found that SCI elevates TLR2, suppresses autophagy (LC3, Beclin-1 ⇣, p62 ⇡), and activates the TLR2/NLRP3/Caspase-1/IL-1β cascade. Red/near-infra-red photobiomodulation downregulated TLR2, restored autophagic markers, curtailed inflammasome activity, and improved hind-limb recovery, implicating TLR2 as a photomodulatable hub linking innate immunity to autophagy (Zuo et al., 2025).

Spared-nerve injury stalled autophagy in dorsal root ganglia macrophages (p62 ⇡, LC3-II, Beclin-1 altered). EA phosphorylated AMPK, inhibited mTOR, normalized autophagic flux, and lessened pain hypersensitivity—highlighting macrophage-rather-than-neuron autophagy as the decisive target of this ancient neuromodulatory technique (Xu et al., 2022).

Running exercise lowered spinal BDNF, thereby quenching AKT/mTOR activity, boosting microglial autophagy, and driving an M1 to M2 phenotypic shift. Autophagy inhibition reversed the analgesic benefit, confirming that exercise-induced autophagy is causal rather than correlative in mitigating neuropathic pain (Bai et al., 2022).

Collectively, physical/neuromodulatory strategies converge on the re-activation of microglial or macrophage autophagy as a mechanistic fulcrum for neuro-protection and pain relief after SCI. Despite diverse energy sources studies documented that impaired autophagic flux is a common pathological denominator following SCI or peripheral nerve injury. Restoration of that flux consistently shifts cytokine output toward an anti-inflammatory profile (IL-10, M2 markers) and attenuates canonical inflammasome signaling (TNF-α, IL-1β, p38 MAPK, TLR2–NLRP3). Up-stream gating molecules differ by modality—GRK2 for HVPRF, TLR2 for photobiomodulation, AMPK for EA, BDNF for exercise—yet all funnel through the mTOR axis, underscoring the pathway’s therapeutic tractability. Future works should integrate multimodal stimulation with precise autophagy reporters, expand to large-animal models, and align dosing regimens with feasible clinical protocols to move these promising biophysical interventions from bench to bedside.

#### 2.3.7 Nanomedicine and multi-drug delivery systems

Moreover, zein-based nanoparticles co-delivering celastrol, metformin, and everolimus restore autophagy in BV2 microglial cells by inhibiting mTOR and enhancing mitochondrial function. Celastrol also activates the nuclear receptor subfamily four group A member 1 (NR4A1; Nur77) pathway, inducing translocation to mitochondria and promoting mitophagy. These effects suppress inflammatory cytokine release (e.g., TNF-α, IL-6), reduce oxidative stress, and protect neurons. Enhanced autophagic activity improves spinal cord tissue repair and motor function in rats, highlighting autophagy modulation in microglia as a therapeutic avenue for SCI (Wang J. et al., 2024).

## 3 Pyroptosis

Pyroptosis is a form of programmed cell death characterized by its inflammatory nature, distinct from apoptosis and necrosis. It is primarily mediated by the activation of inflammatory caspases, notably caspase-1 in the canonical pathway and caspases-4/5 (in humans) or caspase-11 (in mice) in the non-canonical pathway. These caspases cleave GSDMD, releasing its N-terminal fragment, which forms pores in the cell membrane, leading to cell swelling, lysis, and the release of proinflammatory cytokines such as IL-1β and IL-18. The process is typically initiated by the recognition of PAMPs or DAMPs by pattern recognition receptors (PRRs), leading to the assembly of inflammasomes like NLRP3, which then activate the caspases (Al Mamun et al., 2021; Man et al., 2017).

In the context of SCI, pyroptosis has been implicated in exacerbating secondary injury processes. Following the initial mechanical injury, a cascade of inflammatory responses ensues, wherein pyroptosis contributes to the death of neurons and glial cells, amplifying neuroinflammation and tissue damage. Studies have shown that components of the pyroptotic pathway, including NLRP3 inflammasome, caspase-1, and GSDMD, are upregulated in SCI models, suggesting their active role in the pathology. Targeting these molecules to inhibit pyroptosis presents a potential therapeutic strategy to mitigate inflammation and promote recovery in SCI patients (Al Mamun et al., 2021; Liu F. S. et al., 2024).

### 3.1 Molecular and cellular mechanisms

#### 3.1.1 Non-coding RNAs

A growing body of evidence shows that micro- and macro-non-coding RNAs orchestrate the inflammasome machinery that drives microglial pyroptosis after SCI. Most of the work to date converges on a simple paradigm. miRNAs tend to brake pyroptosis, whereas longer non-coding species (lncRNAs and circRNAs) often release that brake—although exceptions exist. Below, the principal studies are arranged to emphasize this continuum from miRNA-mediated restraint to lncRNA/circRNAs-mediated activation.

Resveratrol restores locomotor function after SCI in rats largely by boosting microglial miR-124-3p. The miRNA silences Death-Associated Protein Kinase 1 (DAPK1), uncoupling NLRP3 from its activating scaffold and suppressing Caspase-1/GSDMD cleavage. Genetic loss of DAPK1 or forced miR-124-3p expression phenocopies the drug, cutting IL-1β release and rescuing cell viability (Li D. et al., 2025).

MSC–derived EVs loaded with miR-22 deliver the miRNA directly into microglia. miR-22 pairs with the 3′-UTR of GSDMD, blunting pore formation and the IL-1β/IL-18/TNF-α surge, thereby accelerating neurological recovery beyond that achieved by unmodified EVs (Sheng et al., 2021).

Regulatory T cells (Treg) secrete exosomes enriched in miR-709. Once inside microglia, miR-709 downregulates NF-κB activating protein (NKAP), damping inflammasome priming. Depleting Tregs worsens motor scores, whereas adoptive transfer or purified exosomes reverses the deficit, highlighting a physiological checkpoint on pyroptosis (Xiong et al., 2022).

Switching focus from microglia to their neuronal neighbors, exosomes from M2-polarized microglia ferry miR-672-5p into injured neurons. The target is AIM2, whose repression collapses the AIM2/ASC/Caspase-1 axis, limits neuronal pyroptosis, and supports axon regrowth—an indirect yet important microglia-to-neuron relay (Zhou et al., 2022).

Induced pluripotent stem cell–derived neural stem cells (NSCs) release exosomes rich in let-7b-5p. In microglia, the miRNA represses Leucine-Rich Repeat Inherited Protein 3 (LRIG3), upstream of NLRP3, curtailing GSDMD cleavage and cytokine release. Animals receiving either the cells or isolated exosomes show comparable gains in motor function (Liu J. et al., 2024).

In contrast, SCI upregulates the lncRNA F630028O10Rik *via* Toll-like receptor 4 (TLR4)/Signal Transducer and Activator of Transcription 1 (STAT1) signalling. Acting as a competing endogenous RNA, it sponges miR-1231-5p, de-repressing Collagen Type I Alpha 1 Chain (Col1a1) and activating the PI3K/AKT cascade to fuel microglial pyroptosis. Blocking any node in this axis diminishes cell death and improves outcome (Xu et al., 2020).

The circular RNA circ0000381 sequesters miR-423-3p, indirectly boosting NLRP3 expression. Most data indicate that this escalates inflammasome activation and cytokine release; paradoxically, circ0000381 knock-down was reported to increase pyroptosis in one model, pointing to cell-type or temporal complexities that remain unresolved (Zhang Y. et al., 2024).

Collectively, five independent reports converge on a common protective motif. Exogenous or endogenous miRNAs (miR-124-3p, -22, -709, -672-5p, let-7b-5p) target distinct but convergent nodes (DAPK1, GSDMD, NKAP, AIM2, LRIG3) to dampen the NLRP3 or AIM2 axis. Delivery vehicles range from small molecules that upregulate endogenous miRNA to designer stem-cell or immune-cell exosomes—highlighting translatable platforms already in pre-clinical use.

Conversely, longer non-coding species appear to lift this brake, with lncRNA F630028O10Rik and circ0000381 both relieving repression of pyroptosis-promoting genes. Whether this reflects a general pro-pyroptotic role for lncRNAs/circRNAs or merely injury-timed expression spikes is still uncertain, underscored by the contradictory data on circ0000381.

However, most studies rely on single-sex rodent models and acute time-points. Network cross-talk among multiple ncRNAs is largely unexplored. Off-target and dose-limiting toxicities of exosome therapies remain to be defined. Addressing these issues will determine whether ncRNA-based modulation of microglial pyroptosis can move from bench to bedside.

#### 3.1.2 Transcription factors and epigenetic regulators

Transcription factors and chromatin-modifying enzymes orchestrate the intensity and duration of microglial pyroptosis after SCI. By modulating the expression, activation, or degradation of key inflammasome components, they tilt the balance between neuro-destructive and reparative responses.

SCI rapidly induces the transcription factor CCAAT/Enhancer-Binding Protein Beta (C/EBPβ) in microglia. C/EBPβ binds to the Fc Gamma Receptor I (Fcgr1) promoter, drives its expression, and thereby facilitates NLRP3-inflammasome priming. The ensuing rise in caspase-1 activity and GSDMD cleavage releases IL-1β, IL-18, IL-6 and TNF-α, which propagate neuronal apoptosis and hinder functional recovery. Genetic knock-down of either C/EBPβ or Fcgr1, or pharmacological blockade of caspase-1 with VX-765, breaks this feed-forward loop and improves behavioral outcomes, identifying the C/EBPβ–Fcgr1 axis as a tractable transcriptional target (Li J. et al., 2025).

In a chronic constriction injury model, activated spinal microglia upregulate the cytoplasmic de-acetylase HDAC6. HDAC6 enhances NF-κB nuclear translocation, boosts NLRP3 expression, and accelerates the caspase-1/GSDMD pathway, culminating in IL-18 release and pyroptotic cell death. Selective HDAC6 inhibition with ACY-1215 dampens microglial activation, lessens mechanical allodynia, and highlights epigenetic acetylation status as a switch that gates pyroptotic pain after SCI (Sun et al., 2023).

Conversely, the antioxidant transcription factor nuclear factor erythroid 2-related factor 2 (Nrf2) is suppressed after SCI. Restoring Nrf2 upregulates miR-146a, a microRNA that directly targets the GSDMD mRNA 3′-UTR. Reduced GSDMD translation lowers caspase-1 activation and attenuates IL-1β and IL-18 release. Both *in vitro* and *in vivo*, Nrf2 over-expression preserves microglial viability and limits tissue loss, positioning the Nrf2/miR-146a/GSDMD axis as an endogenous anti-pyroptotic circuit (Zhang et al., 2023).

The E3 ubiquitin ligase Tripartite Motif Containing 32 (TRIM32), downregulated after SCI, affords similar protection when re-introduced. TRIM32 binds the NLRP3 co-factor NIMA-related kinase 7 (NEK7) and catalyzes its K64-linked ubiquitination, targeting NEK7 for proteasomal degradation. With less NEK7 available to license NLRP3 oligomerization, caspase-1 and GSDMD activation fall, limiting IL-1β/IL-18 efflux and tissue damage. Thus, TRIM32 links post-translational ubiquitin editing to inflammasome restraint (Yu et al., 2025).

Together, these studies converge on a unifying theme. Transcriptional and epigenetic checkpoints dictate whether microglia intensify or diffuse pyroptotic signalling after SCI. C/EBPβ and HDAC6 act as pro-pyroptotic “accelerators,” respectively amplifying Fc-receptor transcription and de-acetylating NF-κB to sustain NLRP3 expression. In contrast, Nrf2 and TRIM32 function as “brakes,” suppressing GSDMD translation and NEK7 availability. All four pathways ultimately modulate the same caspase-1/GSDMD pore-forming axis, underscoring its centrality.

Despite clear therapeutic promise, the evidence remains limited to rodent or cellular models, with short observation windows and little insight into temporal cross-talk among these regulators. Whether simultaneous modulation of multiple nodes is synergistic, redundant, or detrimental is unknown, as is the impact on other microglial death modes (e.g., ferroptosis, necroptosis). Future work should integrate time-resolved multi-omics and conditional knockout approaches to map the hierarchy of these checkpoints *in vivo* and to test combination strategies that fine-tune microglial pyroptosis for optimal neurological recovery.

#### 3.1.3 Enzymes, surface molecules and antioxidant systems

After SCI, microglia switch on the NLRP3-caspase-1–GSDMD axis, causing pyroptotic rupture and a surge of IL-1β/IL-18 that fuels secondary tissue loss. A growing body of work shows that apparently unrelated enzymatic or chaperone systems converge on this same pathway and can either dampen or amplify it.

The ecto-enzyme CD73 (ecto-5′-nucleotidase) exemplifies a brake that operates at the cell surface. SCI-induced hypoxia first raises HIF-1α, which transcriptionally upregulates CD73 on microglia. Once expressed, CD73 converts extracellular AMP to adenosine; adenosine engages Adenosine A2B receptor (A2B) receptors and activates PI3K/AKT signalling, which inactivates the transcription factor Forkhead box O1 (FoxO1). FoxO1 would otherwise drive GSDMD gene expression, so its inhibition keeps the NLRP3–GSDMD axis in check. In a positive feedback loop, A2B–p38 MAPK signalling feeds back to sustain HIF-1α and therefore CD73 itself, consolidating suppression of pyroptosis and improving histological and functional outcomes after SCI (Xu et al., 2021).

Ceramide synthase-5 (CerS5) has the opposite effect by rewiring lipid metabolism. CerS5 preferentially generates C16-ceramide, which upregulates Phospholipase A2 Group VII (Pla2g7) and primes NLRP3. Down-stream caspase-1 cleavage of GSDMD produces membrane pores, heightening microglial pyroptosis, neuronal loss and behavioral deficits. Genetic or pharmacological blockade of CerS5 lowers C16-ceramide, silences Pla2g7–NLRP3 signalling and markedly improves recovery, positioning CerS5 as a metabolic accelerator of the pyroptotic response (Zhang et al., 2025).

The inducible heat-shock protein HSPA1A (Hsp70-1A) acts as an intracellular chaperone-mediated shield. HSPA1A upregulates the MAPK phosphatase Dual-Specificity Phosphatase 1 (DUSP1), which dephosphorylates p38, c-Jun N-terminal kinase (JNK) and Extracellular Signal-Regulated Kinases 1 and 2 (ERK1/2). Dampening these stress kinases prevents full assembly of the NLRP3 inflammasome, blocks caspase-1/GSDMD activation and curtails IL-1β/IL-18 releases. In rat SCI models, adeno-associated-virus–driven HSPA1A significantly limits microglial pyroptosis and reduces neuroinflammation, translating into better locomotor scores (He et al., 2025).

Glutathione peroxidase-3 (GPx3) brings the antioxidant arm into play. By detoxifying reactive oxygen species, GPx3 interrupts the ROS-driven activation of Interleukin-1 Receptor-Associated Kinase 4 (IRAK4), a kinase that normally propels the IRAK4/ROS/NLRP3 feed-forward loop. Direct binding of GPx3 to IRAK4 destabilizes the kinase, further quenching inflammasome activity and GSDMD cleavage. Viral over-expression of GPx3 in microglia therefore lessens pyroptosis, dampens cytokine outflow and accelerates motor recovery after SCI (Liu et al., 2025).

CD73 and GPx3 represent extracellular and antioxidant brakes, respectively; HSPA1A provides a chaperone-based checkpoint inside the MAPK network; CerS5 functions as a metabolic accelerator. Together they map an interconnected landscape in which purinergic, lipid, oxidative-stress and protein-folding cues all feed into the same inflammatory death pathway.

While each intervention yielded robust structural and behavioral rescue in rodent models, several gaps remain. The studies rely heavily on single-gene manipulations; systems-level analyses are needed to rule out off-target effects and to capture crosstalk between the modules. Timing differs as CD73 is most effective early (hypoxia phase), whereas CerS5 and GPx3 peak later. Except for CD73, translation-relevant delivery routes (systemic, intrathecal) have not been rigorously tested. Addressing these issues will clarify whether a combination strategy—simultaneously boosting brakes and blocking accelerators—can offer synergistic protection in human SCI.

#### 3.1.4 Pattern-recognition receptors and inflammasome priming

After SCI, damage-associated molecular patterns such as biglycan engage TLR4 on microglia. The receptor signals through Janus Kinase 2 (JAK2)/STAT1, driving transcription of the RNA helicase DEAD-box Helicase 3 X-Linked (DDX3X). DDX3X in turn facilitates assembly of the NLRP3 inflammasome, culminating in caspase-1 activation, GSDMD cleavage and pyroptotic death. Genetic or pharmacological inhibition of TLR4, JAK2/STAT1 or DDX3X lessens pyroptosis and improves locomotor recovery, identifying this linear cascade as a tractable therapeutic axis (Wang et al., 2022).

TLR4/STAT1 signalling also initiates a non-coding-RNA circuit. STAT1 transactivates the LncRNA F630028O10Rik, which sponges miR-1231-5p. Loss of miR-1231-5p derepresses its target Col1a1, thereby engaging the PI3K/AKT pathway and further priming the NLRP3–caspase-1– GSDMD inflammasome. Interrupting any node—TLR4, STAT1, the lncRNA itself, or PI3K/AKT—dampens pyroptosis and yields better functional outcomes after SCI, underscoring the multilayered control that a single PRR can exert through coding and non-coding effectors (Xu et al., 2020).

In sum, while TLR4-centred signalling clearly shapes inflammasome priming after SCI, a more integrated appraisal of the wider Pattern-Recognition Receptor landscape and of chronic-phase pathology will be essential before these insights can be converted into clinically viable interventions.

### 3.2 Therapeutic and experimental interventions

#### 3.2.1 Polyphenols, flavonoids and phenolic acids

A growing body of work shows that plant-derived polyphenols can restrain microglial pyroptosis after SCI by converging on a few recurrent targets. Below, the main studies are arranged from upstream regulators to metabolic fine-tuning to provide a clearer mechanistic thread.

Resveratrol increases miR-124-3p, which in turn suppresses DAPK1. Because DAPK1 promotes NLRP3 assembly, its downregulation breaks the NLRP3/caspase-1/GSDMD axis, curbing pyroptosis and improving locomotor recovery *in vivo* (Li D. et al., 2025).

Taxifolin counters oxidative stress and dampens NLRP3, ASC, caspase-1 and GSDMD by activating the PI3K-AKT survival pathway. The dual antioxidant/anti-pyroptotic action supports axonal regrowth and functional restoration after SCI (Hu et al., 2023).

Kaempferol lowers NADPH oxidase 4-derived ROS, thereby shutting down p38/JNK-MAPK and NF-κB signalling. This cascade prevents NLRP3 activation, reduces IL-1β/IL-18 release and limits microglial pyroptosis, translating into better behavioral scores (Liu Z. et al., 2021).

Cynarin triggers the Nrf2 antioxidant axis, suppressing ROS and secondarily blocking the NLRP3/caspase-1/GSDMD route. Reduced cytokine release and tissue sparing lead to significant gains in locomotor outcomes (Zhang B. et al., 2024).

Celastrol, a triterpenoid often grouped with polyphenols, directly inhibits NF-κB, limiting NLRP3 priming and favoring an M2 (repair-oriented) microglial phenotype. Lower pyroptotic protein levels coincide with smaller lesions and improved motor recovery (Dai et al., 2019).

Urolithin A, a gut-derived ellagitannin metabolite, enhances mitophagy, stabilizes mitochondrial function and cuts mtROS. The intact mitochondria no longer feed NLRP3 activation, keeping caspase-1 and GSDMD in check and facilitating functional repair—an effect abolished by autophagy blockade (Chen et al., 2025).

Lycium barbarum glycopeptide (LbGp) boosts the Fatty Acid Desaturase 1 and 2 in microglia, increasing docosahexaenoic acid (DHA) production. DHA then suppresses MAPK/NF-κB and NLRP3 signalling, limiting pyroptosis and enhancing motor outcomes in SCI models (Jiang et al., 2023).

Collectively, these studies converge on the NLRP3/caspase-1/GSDMD axis as the main regulator of microglial pyroptosis, yet they intervene at distinct checkpoints. miRNA regulation (resveratrol), canonical survival signalling (taxifolin), redox balance (kaempferol, cynarin), transcriptional priming (celastrol), organelle quality control (urolithin A) and lipid-mediated immunomodulation (LbGp). The consistency of behavioral benefits across compounds strengthens the overall case. However, several limitations remain. Most agents ultimately dampen NLRP3; whether combinations yield additive benefits or plateau remains untested. Few studies address pharmacokinetics or delayed treatment windows, critical for clinical translation. Microglia are the stated target, yet off-target effects on neurons, astrocytes and peripheral immune cells are seldom quantified. Urolithin A and LbGp underscore microbiota or dietary lipid involvement, but causal links in human SCI are unknown.

#### 3.2.2 Stem-cell, conditioned-medium and exosome-based therapies

Microglial pyroptosis becomes a decisive secondary insult after SCI, and several stem-cell–derived secretomes now converge on this target. Collectively, these approaches work by ferrying regulatory non-coding RNAs or other paracrine factors that quench inflammasome activity, blunt cytokine release and create a permissive milieu for axonal regrowth.

The simplest intervention is the whole conditioned medium. Human dental-pulp stem-cell secretome dampens the canonical NLRP3-caspase-1-IL-1β axis in LPS-primed BV2 microglia and in contused rats, translating to reduced neuroinflammation, enhanced axonal sprouting and better locomotor scores (Liu T. et al., 2024).

Going a step further, exosomes from bone-marrow MSCs were loaded with miR-22. Because miR-22 directly targets GSDMD, the executioner of pyroptosis, the engineered vesicles curtailed pore formation and cytokine release, markedly improving behavioral recovery in rats (Sheng et al., 2021).

Olfactory mucosa MSC exosomes traffic the lncRNA RMRP into microglia. RMRP complexes with Eukaryotic Translation Initiation Factor 4A3 (EIF4A3) to stabilize Sirtuin 1 mRNA; elevated Sirtuin 1 then represses NLRP3, caspase-1 and GSDMD, easing motor deficits in mice (Wang C. et al., 2025).

Induced-pluripotent NSCs and their vesicles shuttle let-7b-5p, which silences the adaptor LRIG3, again throttling NLRP3/caspase-1/GSDMD signalling. The result is preserved myelin, denser axonal tracts and superior motor coordination (Liu J. et al., 2024).

Pyroptosis peaks around day 7 post-injury, but endogenous Treg cells temper this surge. Their exosomes deliver miR-709, which knocks down NKAP and thereby the NF-κB feed-forward loop; genetic ablation of Tregs or miR-709 exacerbates, and supplementation ameliorates, sensorimotor decline (Xiong et al., 2022).

Finally, anti-inflammatory M2 microglia lend support to neighboring neurons: their vesicles are enriched in miR-672-5p, an inhibitor of the AIM2/ASC/caspase-1 pathway. Loss of miR-672-5p nullifies the vesicles’ protection, confirming a neuron-centered pyroptosis checkpoint distinct from NLRP3 (Zhou et al., 2022).

Across diverse cell sources (dental pulp, mesenchyme, olfactory mucosa, induced pluripotent cells, Tregs and even homeostatic M2 microglia) the common therapeutic denominator is delivery of RNA cargo that throttles one of three pressure points in the pyroptotic cascade: (i) inflammasome activation (NLRP3 or AIM2), (ii) execution (GSDMD) or (iii) transcriptional priming (NF-κB/NKAP). These studies consistently report reduced cytokine storms and improved locomotion, reinforcing pyroptosis as a tractable target.

Yet several gaps remain. All work is confined to acute or subacute rodent models; chronic lesions, large mammals and female subjects are largely absent. Cargo heterogeneity, isolation protocols and dosing schedules are rarely standardized, hampering cross-study comparisons. Long-term biodistribution, off-target immunomodulation and potential crosstalk with other death axes (ferroptosis, necroptosis) are also untested. Finally, while most strategies harness non-coding RNAs, only one exploits direct GSDMD inhibition—suggesting unexplored space for combinatorial or small-molecule co-packaging. Overall, stem-cell–and immune-cell–derived secretomes offer a multi-modal, low-immunogenic platform to subdue microglial pyroptosis, but rigorous optimization and head-to-head validation are essential before clinical translation.

#### 3.2.3 Physical and metabolic interventions

Segmental (Jiaji) EA delivered soon after SCI reverses microglia-driven pyroptosis and accelerates locomotor recovery. In rat models, EA suppressed the entire NLRP3/caspase-1/GSDMD axis, lowering IL-1β and IL-18 release and shrinking the population of GSDMD-positive microglia in the injured cord. Administration of an NLRP3 agonist partially restored pyroptotic signalling and motor deficits, confirming that EA’s benefit is tightly coupled to inflammasome inhibition rather than to general neurostimulation (Mei et al., 2024).

Extending those findings to chronic neuropathic pain, a second study showed that EA applied at hind-limb acupoints likewise downregulated NLRP3, cleaved caspase-1, N-GSDMD and downstream cytokines—but only in microglia, not neurons or astrocytes. Pharmacological blockade of NLRP3 with MCC950 reproduced EA’s antiallodynic effect and provided no additional benefit when combined, underscoring the specificity of the pathway (Kui et al., 2025).

Intermittent metabolic stress also curbs microglial pyroptosis. Every-other-day Fasting elevated circulating bile acids that engage the G-protein-coupled bile acid receptor 1 (TGR5), a known negative regulator of NLRP3. Activation of TGR5 dampened microglial activation, reduced caspase-1 cleavage and GSDMD pore formation, and, in turn, blunted IL-1β/IL-18 release, limiting secondary tissue loss and improving behavioral outcomes after SCI (Song et al., 2024).

Conversely, exposure to advanced oxidation protein products—biomarkers of systemic oxidative stress—worsened SCI pathology by driving NADPH oxidase-derived ROS production. ROS activated the MAPK/NF-κB signalling cascade, primed the NLRP3 inflammasome and precipitated full-blown microglial pyroptosis. The NADPH oxidase inhibitor apocynin broke this feed-forward loop, attenuating ROS accumulation, suppressing inflammasome formation and improving histological as well as functional outcomes (Liu et al., 2020).

Across diverse pre-clinical settings, both physical (like EA) and metabolic (intermittent fasting, redox manipulation) interventions converge on a single molecular hub—the NLRP3/caspase-1/GSDMD inflammasome—highlighting its centrality in microglial pyroptosis after SCI. EA provides tunable, region-specific suppression of this axis without systemic metabolic perturbation, whereas Every-other-day Fasting offers a non-invasive systemic strategy that leverages endogenous bile-acid signalling. The advanced oxidation protein products (AOPPs) study usefully reminds us that background oxidative stress can push the same pathway in the opposite direction, and that pharmacological antioxidants such as apocynin may be necessary adjuncts.

Strengths of the section include mechanistic depth (each study maps a clear causal chain) and functional read-outs that tie molecular changes to motor or pain phenotypes. Limitations are equally clear: all data are rodent-based, head-to-head comparisons of intervention timing and dosing are missing, and none of the studies examine long-term safety or combinatorial effects with standard neuro-rehabilitation. Future work should (i) validate these approaches in large-animal or humanized models, (ii) dissect potential cross-talk with Ferroptotic and necroptotic pathways, and (iii) explore whether physical and metabolic strategies can be synergistically layered to achieve durable neuroprotection without off-target immune suppression.

#### 3.2.4 Other therapies

Lupenone is a triterpenoid with anti-inflammatory properties and significantly improves SCI outcomes by targeting microglial pyroptosis. In SCI, microglia become overactivated (M1 phenotype), triggering inflammation through the NF-κB/NLRP3/caspase-1/GSDMD axis, leading to pyroptosis. Lupenone inhibits NF-κB activation (*via* p65/IκBα modulation), reducing NLRP3 inflammasome activation, IL-1β/IL-18 releases, and promoting M2 microglial polarization. This dual modulation of pyroptosis and phenotype shift leads to neuroprotection and improved motor recovery (Li F. et al., 2024).

Kanglexin is a novel synthetic anthraquinone, ameliorates SCI by modulating neuroinflammation in microglial cells. Kanglexin inhibits pyroptosis by blocking the NLRP3 inflammasome pathway. It enhances Protein Kinase A (PKA) phosphorylation, which suppresses NF-κB activation and reduces transcription of inflammasome components. Kanglexin also promotes microglial polarization from proinflammatory M1 to anti-inflammatory M2 phenotypes, aiding neurorepair. Overall, Kanglexin protects neural tissue post-SCI by suppressing pyroptosis and shifting microglial phenotype *via* the PKA/NF-κB signaling axis (Yan et al., 2025).

In spinal cord ischemia/reperfusion (SCIR) injury, pyroptosis alongside apoptosis and necroptosis, contributes to neural death. Melatonin, a hormone synthesized in the pineal gland with antioxidant and anti-inflammatory properties, reduces microglial pyroptosis *via* melatonin receptor (MT1/MT2)-mediated signaling. Specifically, melatonin inhibits NLRP3, caspase-1, and GSDMD activation, reducing IL-1β release in microglia. Luzindole, a melatonin receptor antagonist, negates this effect, confirming receptor involvement. Melatonin thereby improves neurological outcomes by suppressing PANoptosis—including microglial pyroptosis—in SCIR rats (Xie et al., 2024a).

## 4 Ferroptosis

### 4.1 Molecular and cellular mechanisms

#### 4.1.1 Ferroptosis and inflammatory regulation

After SCI, activated microglia accumulate iron and release NO that destabilizes neuronal iron homeostasis by up-regulating IRP1, Divalent Metal Transporter 1 (DMT1) and Transferrin Receptor 1 while down-regulating ferritin. The ensuing neuronal iron overload—together with mitochondrial shrinkage and altered Prostaglandin-Endoperoxide Synthase 2 (PTGS2)/Gpx4 expression—triggers neuronal ferroptosis and limits motor recovery. Iron chelators (like deferoxamine), ROS scavengers (like N-acetylcysteine) and the canonical ferroptosis inhibitor ferrostatin-1 all attenuate this cascade, underscoring the central role of microglia-driven iron dysregulation in SCI pathology (Feng et al., 2021).

Ferroptotic microglia themselves amplify inflammation. Their lipid-peroxidation-induced mtROS activate PKR-like endoplasmic reticulum kinase (PERK) signalling, whereas NF-κB drives IL-1β and IL-6 transcription. Together, these events markedly upregulate IL-23, fostering differentiation of pathogenic T helper 17 (Th17) cells that exacerbate tissue damage. Dual inhibition of ferroptosis and IL-23 therefore improves functional recovery in experimental models (Zhao et al., 2024).

TNF-α sits at the core of an iron–TNF–ferroptosis–inflammation axis. Iron overload raises microglial TNF-α, which in turn potentiates lipid peroxidation (4-HNE), depletes glutathione and pushes cells toward an iNOS^+^/CD86^+^ M1 phenotype. Genetic deletion of TNF-α—or pharmacological zinc supplementation—breaks this feed-forward loop, diminishes ferroptosis and shifts microglia toward an Arg1^+^/CD206^+^ reparative state, improving neuronal survival and locomotion (Zeng et al., 2024).

Glycosylated lysosomal membrane protein (GLMP) is an intrinsic brake on ferroptosis. SCI lowers GLMP expression; loss-of-function increases microglial iron deposition, raises COX-2 and acyl-CoA synthetase long-chain family member 4 (ACSL4), damages mitochondria and worsens behavioral outcomes. Conversely, preserving GLMP limits ferroptosis and supports tissue repair, highlighting lysosomal iron handling as a therapeutic entry point (Ouyang et al., 2025).

Epigenetic control further modulates the Ferroptotic response. Protein arginine methyl-transferase-8 (PRMT8) catalyzes Histone 3 Lysine 4 (H3K4) trimethylation to boost GDNF expression. PRMT8 over-expression reduces iron, 4-HNE and Heme Oxygenase 1 (HO-1), curtails ferroptosis and promotes Arg1-dominated M2 microglial polarization, collectively protecting the injured cord (Zou et al., 2023).

Systemic metabolic cues intersect with local Ferroptotic signalling. SCI-induced oxidative stress increases ApoB-100/SORT1 expression, elevating ox-LDL, driving M1 activation and aggravating neuronal ferroptosis. Silencing ApoB-100/SORT1 restores GPX4 and Transferrin Receptor 1, lowers ROS and skews microglia away from an inflammatory phenotype. Intriguingly, M1 macrophages can transiently suppress ferroptosis *in vitro* yet promote it *in vivo*, illustrating the context-dependence of immune–metabolic cross-talk (Wu et al., 2024b).

Collectively, these studies converge on a unifying theme. Iron-laden, lipid-peroxidized microglia are both victims and amplifiers of ferroptosis after SCI. Up-stream regulators range from classical cytokines (TNF-α) and stress pathways (PERK, NF-κB) to lysosomal (GLMP), epigenetic (PRMT8) and metabolic (ApoB-100/SORT1) checkpoints, all feeding into a self-propagating loop of iron accumulation, oxidative damage and inflammatory polarization.

Two conceptual tensions remain. First, microglia can simultaneously incite neuronal ferroptosis and undergo it themselves, but whether they die or survive long enough to orchestrate chronic inflammation is still unclear. Second, the *in-vitro versus in-vivo* discrepancy in M1-mediated ferroptosis points to stage-specific or micro-environmental modifiers that are largely unmapped.

Therapeutically, single-target interventions (iron chelators, ferrostatin-1) show partial benefit, whereas combined strategies—modulating iron handling (GLMP), lipid metabolism (ApoB-100/SORT1) and inflammatory signalling (TNF-α, IL-23)—offer a more compelling rationale. Future work should therefore focus on the following factors: (i) temporal mapping of ferroptosis relative to microglial phenotypic shifts; (ii) multi-omic integration to resolve micro-environmental heterogeneity; and (iii) combinatorial or stage-tailored treatments that can interrupt the ferroptosis–inflammation feedback with greater precision.

### 4.2 Therapeutic and experimental interventions

#### 4.2.1 Small-molecule inhibitors and natural compounds

After SCI, microglia engulf erythrocytic debris and, through HO-1 induction and NCOA4-dependent ferritinophagy, accumulate catalytically active iron that precipitates glutathione (GSH) depletion, lipid peroxidation, and Ferroptotic death. The lipophilic antioxidant UAMC-3203 interrupts this cascade: by restoring GSH and GPX4 activity it limits lipid peroxidation in microglia/macrophage populations, curtails secondary tissue loss, and accelerates locomotor recovery in mice (Ryan F. et al., 2024).

Ferrostatin-1, the prototypical ferroptosis blocker, likewise reduces ROS and iron overload after SCI. Consequent downregulation of the ferroptosis markers iron-responsive element-binding protein 2 (IREB2) and PTGS2 in oligodendrocytes and microglia preserves myelin integrity, dampens gliosis, and improves hind-limb function in rat models (Ge et al., 2022).

A second study with the more water-soluble analog UAMC-3203 confirms and extends these findings: activation of the Nrf2/HO-1 antioxidant axis normalizes GPX4, and GSH levels, while simultaneously re-balancing the inflammatory milieu—suppressing TNF-α and IL-1β and boosting IL-4 and IL-10. This dual ferroptosis-modulating and immunoregulatory action protects neurons and further enhances behavioral recovery (Kan et al., 2024).

Punicalagin, a pomegranate-derived polyphenol, targets the same Nrf2/solute carrier family 7 member 11 (SLC7A11)/GPX4 axis but from a natural-compound vantage. By preventing iron-dependent lipid peroxidation in neurons and driving microglia toward the reparative M2 phenotype, it synchronously attenuates oxidative stress and fosters axonal regrowth, resulting in superior functional outcomes (Wang W. et al., 2025).

Albiflorin, isolated from *Paeonia lactiflora*, inhibits lysine-specific demethylase-1 (LSD1). Because LSD1 upregulation promotes ferroptosis, its suppression by Albiflorin lowers lipid peroxidation and iron accumulation in microglia, dampens proinflammatory cytokine release, and supports motor recovery (Zhang L. et al., 2024).

Gallic acid, another plant phenolic, mitigates chronic post-SCI pain and depression by restraining P2X purinoceptor 7 (P2X7)-driven TNF-α/TNF-α–converting enzyme (TACE; ADAM17)/NF-κB/STAT3 signalling. The downstream result is reduced ROS, malondialdehyde, and iron load, restored GSH/GPX4, preserved mitochondrial morphology, and ultimately less microglial ferroptosis and neuroinflammation (Yang R. et al., 2023).

Across small synthetic inhibitors (Ferrostatin-1, UAMC-3203) and phytochemicals (punicalagin, Albiflorin, gallic acid) a convergent therapeutic principle emerges: preventing iron-catalyzed lipid peroxidation while re-establishing the antioxidant (GSH–GPX4) axis halts microglial ferroptosis and its inflammatory sequelae, translating into robust neuro-protection and functional improvement. Notably, UAMC-3203’s superior solubility and its additional immunomodulatory effects position it as a leading candidate for clinical translation. Natural compounds broaden the armamentarium and—by engaging epigenetic (like LSD1) or purinergic (like P2X7) nodes—highlight novel ancillary targets. Yet most studies rely on acute rodent models; long-term efficacy, optimum dosing, and combinatorial potential with existing neuro-rehabilitative strategies remain open questions demanding systematic investigation.

#### 4.2.2 Nanomedicine and biomaterial platforms

A growing body of work shows that well-designed nanomaterials can temper microglia-driven ferroptosis and thereby limit secondary damage after SCI. Although each platform exploits a different carrier, all converge on three anti-Ferroptotic principles: (i) lower the free Fe^2+^ pool, (ii) quench iron-driven lipid peroxidation, and (iii) re-program microglia toward a reparative (M2-like) phenotype. Below, the studies are arranged from the most targeted genetic approach to multi-functional drug-delivery systems, highlighting the incremental design logic that runs through the field.

Macrophage-targeted phosphatidylserine-lipid nanoparticles (PS/LNPs) carrying *Mms6* mRNA give injured spinal cords an “endogenous magnetite factory.” Translated Mms6 protein nucleates magnetite cores that trap free iron, lowering the Fe^2+^ pool and lipid peroxidation. Selective PS-mediated uptake restricts expression to microglia/macrophages; when these cells are experimentally depleted, the motor and histological gains disappear—proving that ferroptosis control in this compartment is central to recovery (Fu et al., 2025).

An injectable carboxymethyl-cellulose/quaternised-chitosan hydrogel embedded with polydopamine nanoparticles (CQP hydrogel) chelates Fe^2+^ and scavenges ROS directly inside microglia. By re-activating the GPX4 lipid-repair axis, the gel attenuates ferroptosis and simultaneously tilts microglia from an M1 to M2 state, culminating in reduced lesion volume and improved locomotion (Shi et al., 2024).

Mesoporous selenium nanoparticles pre-loaded with resveratrol and cloaked in borneol-modified macrophage membranes cross the BSCB and dock onto resident microglia. Selenium-triggered GPX-like activity, together with resveratrol, raises GPX4/SOD, increases GSH, depresses malondialdehyde (MDA), and curbs ROS. The dual redox hit blunts ferroptosis, dampens inflammation and apoptosis, and translates into robust motor recovery (Liu X. et al., 2024).

Ferrostatin-1/selenium/ZIF-8 nanoparticles wrap selenium cores in zeolitic imidazolate framework-8 (ZIF-8) and ferrostatin-1. The hybrid particle scavenges ROS, safeguards mitochondria, and delivers a canonical ferroptosis inhibitor in one shot. As ferroptosis markers fall, microglia/macrophages polarize toward an anti-inflammatory M2 phenotype, inflammatory cytokines wane, and spared neurons support behavioral gains (Zhou et al., 2024).

A paclitaxel-idebenone prodrug self-assembles into nanomicelles that home to chondroitin-sulphate-rich lesion matrices. Idebenone restores the ferroptosis suppressor protein 1 (FSP1) ferroptosis-suppression pathway and curbs 4-HNE lipid adducts, while paclitaxel stabilizes microtubules to guide axon regrowth. The combined anti-Ferroptotic and pro-regenerative actions reduce microglial activation, preserve neurons, and enhance functional recovery (Xu Z. et al., 2024).

Collectively, these platforms prove that nanomedicine can hit multiple ferroptotic nodes simultaneously while steering microglial phenotype—something single-target small molecules rarely achieve.

Yet several limitations persist. Heterogeneous read-outs. Each study tracks a different slice of the ferroptosis pathway (GPX4, FSP1, Fe^2+^, 4-HNE, *etc.*), making head-to-head efficacy comparisons impossible. A consensus biomarker panel is urgently needed.

Cell-type specificity. Most reports infer microglial targeting from bulk tissue analyses or macrophage depletion, but single-cell RNA/proteomics would verify true selectivity and off-target risks. Selenium, magnetite, and ZIF-8 scaffolds can accumulate; only short-term biosafety is shown. Chronic retention and systemic redistribution remain open questions. Clinical translatability. Injectable hydrogels and PS/LNPs are already close to regulatory paradigms, whereas membrane-coated or metal-organic cages face sterility, scale-up, and reproducibility hurdles. The field is converging on multifunctional “all-in-one” particles, yet additive complexity may outpace manufacturing and regulatory capacity. Rational minimalism—identifying which ferroptotic lever truly dictates outcome—could streamline the path to the clinic.

In summary, nanoplatforms that couple iron detoxification with lipid-repair and immune re-balancing offer a compelling anti-ferroptotic strategy for SCI, but rigorous comparative studies and long-term safety profiling are essential before clinical translation.

## 5 Necroptosis

### 5.1 Molecular mechanisms and therapeutic interventions

Necroptosis emerges soon after SCI in several resident and infiltrating cell types. Mounting evidence positions microglia at the hub of this response: they not only undergo necroptosis themselves but also propagate it to neighboring neural, endothelial and stem-cell compartments through cytokine and vesicular routes. Below, the key mechanistic nodes and intervention strategies are surveyed.

Endoplasmic reticulum stress couples metabolic overload to microglial necroptosis. Post-traumatic microglia/macrophages upregulate Receptor-Interacting Protein Kinase 3 (RIP3) and MLKL, which colocalize with the endoplasmic reticulum chaperone GRP78. *In vitro*, oxygen–glucose deprivation reproduces this endoplasmic reticulum stress signature, whereas the chemical chaperone 4-phenylbutyrate dampens both GRP78 induction and necroptotic execution. These findings place the stressed endoplasmic reticulum upstream of RIP3/MLKL activation and identify endoplasmic reticulum stress modulators as tractable therapeutic leads (Fan et al., 2015).

Microglial TNF-α propagates necroptosis to the vascular niche, but Vagus-nerve stimulation (VNS) breaks the loop. Activated microglia flood the lesion with TNF-α, which in turn drives endothelial RIP1/RIP3/MLKL activation, disrupts the BSCB and amplifies leukocyte infiltration. VNS suppresses microglial TNF-α release, blocks endothelial necroptosis, preserves BSCB integrity and markedly improves locomotor recovery, illustrating how remote neuromodulation can intercept a microglia-vascular necroptotic axis (Chen et al., 2023).

Necroptotic NSCs reshape microglial behavior through exosomal Tuberous Sclerosis Complex 2 (TSC2). Ependymal-derived NSCs succumb to TNF-mediated necroptosis after SCI but remain biologically active by exporting intact exosomes that are reprogrammed to deliver TSC2 mRNA. Uptake of these vesicles by peri-lesional cells boosts TSC2, restrains mTOR signalling and is predicted to favors autophagy-mediated debris clearance. Single-cell communication maps reveal that microglia both initiate NSC necroptosis and respond to TSC2-rich exosomes *via* epidermal growth factor pathways, hinting at a negative-feedback circuit that tempers chronic inflammation (Li S. et al., 2025).

Quercetin deflects microglial polarization and safeguards oligodendrocytes from necroptosis. The flavonoid quercetin skews microglia away from an M1-like phenotype by inhibiting STAT1 and NF-κB, curbing production of iNOS and TNF-α. Lower cytokine pressure translates into reduced RIP3/MLKL activation in oligodendrocytes, improved myelin preservation and superior functional recovery. Quercetin therefore exemplifies a small-molecule strategy that indirectly blocks necroptosis by re-educating its principal instigator—microglia (Fan et al., 2019).

Collectively, these studies converge on a microglia-centered model of necroptosis that radiates to endothelial cells, neural stem cells and oligodendrocytes, thereby amplifying secondary pathology after SCI. Two mechanistic nodes—endoplasmic reticulum stress and TNF-α/RIP-kinase signalling—emerge as common denominators, whereas intercellular communication relies on soluble cytokines, autonomic input and exosome cargo re-programming. Therapeutically, the portfolio already spans a chemical chaperone (4-PBA), a neuromodulatory approach (VNS) and a nutraceutical (quercetin). Each attenuates necroptosis through distinct yet partially overlapping routes, suggesting that combination regimens could achieve additive or synergistic protection.

However, the evidence is almost entirely rodent-based, and cell-type specificity is inferred largely from marker colocalization rather than genetic fate-mapping. Dose, timing and off-target profiles of systemic agents such as 4-PBA or quercetin are insufficiently characterized for clinical translation. Moreover, necroptosis is rarely studied in the chronic phase, leaving its contribution to long-term neuro-degeneration and pain unresolved. Future work should integrate cell-selective inhibition, longitudinal behavioral read-outs and assessment of human SCI tissue to clarify causality and therapeutic windows.

## 6 PANoptosis

### 6.1 Molecular mechanisms and therapeutic interventions

PANoptosis is an integrated cell-death axis that weaves together pyroptosis, apoptosis, and necroptosis through a multi-protein scaffold—the PANoptosome—most often built around the nucleic-acid sensor Z-DNA-binding protein 1 (ZBP1). In the injured spinal cord, activated microglia/macrophages are now recognized as a key cellular compartment in which PANoptosis can either amplify secondary damage or, when restrained, curb the inflammatory cascade.

The anti-cancer curaxin CBL0137 intercalates into deoxyribonucleic acid (DNA) and flips B-DNA (right-handed double helix form of DNA) into the left-handed Z-conformation. Z-DNA is detected by ZBP1, which nucleates a PANoptosome containing RIPK3, caspase-8, and ASC, culminating in the activation of caspases-1/3/8/9, cleavage of GSDMD/GSDME, and phosphorylation of MLKL. This NLRP3-independent execution causes systemic inflammation and multi-organ injury in mice, placing CBL0137-induced PANoptosis squarely within the pathophysiological spectrum of SCI (Li YP. et al., 2024).

The flavone baicalin (Scutellaria baicalensis) shields mitochondria, prevents the release and Z-conversion of oxidized mitochondrial DNA (mtDNA), and thereby aborts ZBP1 recruitment of RIPK3, ASC, and caspase-8. By dismantling this upstream node, baicalin attenuates pyroptotic signalling in microglia/macrophages and suppresses neuroinflammation, suggesting a tractable route to PANoptosis inhibition in SCI (You et al., 2025).

Scutellarin (Erigeron breviscapus) acts along a similar mitochondrial axis. By quenching mtROS, it forestalls PANoptosome assembly (ASC, RIPK3, caspase-8, ZBP1, p-MLKL) *in vitro* and *in vivo*, again without relying on NLRP3 or caspase-1. The result is dampened systemic inflammation and preserved organ integrity after SCI-relevant challenges (Yuan et al., 2024).

Endogenous melatonin, *via* its melatonin receptors and downstream PI3K/AKT signalling, inhibits the NLRP3/caspase-1/GSDMD/IL-1β axis in spinal cord ischemia/reperfusion. Luzindole abrogates this effect, underscoring the receptor dependence of melatonin’s anti-PANoptotic action in microglia and neurons (Xie et al., 2024a).

The slow H_2_S donor GYY4137 provides a multi-hit blockade. It suppresses NLRP3 activation, caspases-1/3/8, pro-apoptotic Bax/Bad, and RIP1/RIP3/MLKL phosphorylation while limiting GSDMD cleavage and M1 polarization. These converging effects lessen neuronal loss and improve locomotor recovery after SCIR injury (Xie et al., 2024b).

Collectively, these studies position PANoptosis as a central hub in microglia-driven secondary pathology after SCI. CBL0137 functions as a trigger, confirming that ZBP1-centred PANoptosomes can single-handedly propel systemic inflammation. In contrast, baicalin and scutellarin exploit the same ZBP1 gateway from the opposite direction, whereas melatonin and GYY4137 intervene further downstream, tuning inflammasome or necroptotic arms. The convergence on mitochondrial stress and Z-DNA formation emerges as a recurrent therapeutic lever, yet the field still leans on acute, often pharmacological, models. Future work must clarify long-term impacts on neuro-regeneration, resolve potential off-target immunosuppression, and test combinatorial regimens that synchronize mitochondrial protection with precise checkpoint inhibition inside the PANoptosome.

Recent works suggest that PANoptosis can be identified by the concurrent activation of pyroptotic, apoptotic, and necroptotic arms within the same cellular context. Candidate molecular markers therefore include the upstream nucleator ZBP1 and the inflammasome adaptor ASC, both of which scaffold PANoptosome assembly. Downstream, the caspase-8–RIPK3–MLKL axis indicates necroptotic engagement, while caspase-1 activation and GSDMD cleavage represent the pyroptotic component, and caspase-3/9 activity reflects the apoptotic contribution. Simultaneous detection of these molecules—particularly co-expression of ZBP1, caspase-8, caspase-1, RIPK3, and phosphorylated MLKL—may thus serve as a molecular signature of PANoptosis in microglia after SCI. Establishing such a marker panel would allow distinction of PANoptosis from isolated death pathways and provide a framework for targeted therapeutic interventions.

## 7 Integrated crosstalk

In this section, we have discussed how autophagy, pyroptosis, ferroptosis, necroptosis, and PANoptosis interlock to shape microglial responses after SCI. These pathways converge at shared regulators reciprocally amplify mixed-death execution. Autophagy (*via* mitophagy, lipophagy, and TFEB-lysosome competence) acts as a context-dependent brake or feed-forward amplifier by routing inflammasome components, ferritin/iron handling, and damaged organelles. This network logic explains why blocking one arm can blunt PANoptotic and necroptotic spread, whereas autophagy failure primes multiple death programs simultaneously. Framed this way, phase-specific, combinatorial strategies—early mitochondrial/lysosomal repair with selective inflammasome or iron-lipid repression—offer the best leverage to collapse mixed-death amplification while preserving reparative microglial states. Table 1 summarizes the crosstalk between these cel death pathways.

**TABLE 1:** Summary of integrated crosstalk among PANoptosis, pyroptosis, ferroptosis, necroptosis, and autophagy in SCI.

Interaction	Core mechanisms	Upstream/downstream regulators	Therapeutic implications
PANoptosis ↔ Pyroptosis	PANoptosis incorporates pyroptotic arm *via* caspase-1 → GSDMD/GSDME cleavage; ZBP1–PANoptosome recruits ASC, caspase-8, RIPK3 → shared execution with apoptotic and necroptotic effectors.	Mitochondrial stress (oxidized mtDNA, mtROS); Z-DNA → ZBP1; NLRP3–caspase-1–GSDMD axis.	Mitophagy/autophagy restores balance; inhibitors (melatonin, GYY4137, baicalin, scutellarin) reduce pyroptosis and PANoptosis simultaneously.
Ferroptosis ↔ Autophagy	Ferritinophagy (NCOA4) releases labile iron → lipid peroxidation; lysosome competence (TFEB, GLMP) brakes ferroptosis; ferroptosis feeds back to trigger autophagy.	HO-1, NF-κB, ACSL4, COX-2; lysosomal integrity; mitophagy lowering mtROS.	Ferroptosis inhibitors (Fer-1, UAMC 3203) + autophagy restoration (TFEB activation, mitophagy) collapse iron–lipid feedback and protect against injury.
Pyroptosis ↔ Autophagy	Autophagy degrades NLRP3 inflammasome, lowers mtROS *via* mitophagy; feedback loop where inflammasome activity suppresses autophagy.	AMPK/ULK1, PINK1–Parkin, TFEB/lysosome; MCC950 (NLRP3 inhibition); CD36 blocks lipophagy.	Autophagy inducers (rapamycin, trehalose, CB2 agonist JWH-133, zinc, urolithin A, ginkgolide B) suppress NLRP3–caspase-1–GSDMD signaling and improve recovery.
Autophagy ↔ Necroptosis	Autophagy disruption (lysosomal dysfunction, mTOR hyperactivation) → RIPK1/RIPK3 accumulation → necroptosis; necroptotic NSCs export TSC2 mRNA to restore autophagy.	ER stress co-localizes with RIPK3/MLKL; AMPK–mTOR–Beclin-1 axis; exosomal TSC2 restrains mTOR.	Autophagy induction (rapamycin, resveratrol, AMPK activators) reduces necroptosis; TSC2-based exosome therapy proposed for rebalancing.
Necroptosis ↔ Ferroptosis	Iron overload + lipid peroxidation elevate TNF-α, fueling RIPK1/RIPK3/MLKL necroptosis; ER stress from ferroptosis converges on necroptosis.	TNF-α loop; NF-κB activation; mtROS; PERK–ER stress; 4-HNE.	Dual targeting: iron chelation, GPX4 restoration, antioxidant nanoplatforms + TNF-α suppression or ER stress inhibitors (e.g., 4-PBA) block both ferroptosis and necroptosis.

### 7.1 PANoptosis and pyroptosis

PANoptosis and pyroptosis are tightly intertwined in SCI microglia. PANoptosis incorporates the pyroptotic arm *via* caspase-1 activation and GSDMD/GSDME cleavage. It can be triggered by mitochondrial and nucleic-acid stress upstream of inflammasomes or independently of NLRP3. Interventions that suppress the NLRP3–caspase-1–GSDMD axis also attenuate PANoptotic execution *in vivo*. The ZBP1-centered PANoptosome serves as an upstream scaffold that converges on pyroptotic machinery alongside apoptotic and necroptotic modules, creating a shared execution cascade that amplifies inflammation unless checked by mitophagy/autophagy and targeted inhibitors.

The connection between PANoptosis and pyroptosis is explicit, as ZBP1-driven PANoptosome assembly recruits RIPK3, caspase-8, and ASC, culminating in the activation of caspases-1, -3, -8, and -9 with GSDMD/GSDME cleavage. This process embeds pyroptotic pore formation within the broader PANoptotic program and links it to necroptotic MLKL phosphorylation in the same execution burst. PANoptosis can proceed in an NLRP3-independent manner, such as in the case of CBL0137 → Z-DNA → ZBP1 PANoptosome, yet still activates caspase-1 and GSDMD. This demonstrates that pyroptotic effectors are integral to PANoptosis, even when classical inflammasome priming is bypassed.

Mitochondrial stress sits upstream of both pathways. Oxidized mtDNA release and Z-conversion promote ZBP1 recruitment and PANoptosome assembly, while mitochondrial reactive oxygen species (mtROS) prime pyroptosis. Blocking these events with baicalin or scutellarin prevents ZBP1 complex formation and attenuates pyroptotic signaling in microglia and macrophages. Conversely, restoring mitophagy/autophagy reduces mitochondrial danger signals, disassembles ASC specks, and diminishes caspase-1 activation, thereby dampening both pyroptosis and the PANoptotic escalation it seeds under persistent stress.

The pyroptotic arm within PANoptosis is defined by caspase-1 activation and GSDMD cleavage, producing IL-1β/IL-18 release. PANoptosis layers apoptotic (caspases-3, -8, -9) and necroptotic (RIPK3-p-MLKL) effectors onto the same episode, which explains the heightened inflammatory amplitude relative to isolated pyroptosis. Because the PANoptosome contains ASC and can activate caspase-1, inflammasome-linked executors become shared nodes, creating a crosstalk interface. This interface allows classical pyroptotic inhibitors to partially blunt PANoptotic damage.

Anti-pyroptotic interventions exhibit anti-PANoptotic effects. For instance, melatonin reduces the NLRP3/caspase-1/GSDMD/IL-1β axis *via* MT1/MT2–PI3K/AKT signaling and is described as anti-PANoptotic in SCI models, indicating that suppressing the pyroptotic arm constrains PANoptosis *in vivo*. The slow H2S donor GYY4137 suppresses NLRP3, caspases-1, -3, -8, RIP1/3, and MLKL, while limiting GSDMD cleavage and M1 polarization. This demonstrates coordinated down-tuning of pyroptotic and PANoptotic (and necroptotic/apoptotic) modules through overlapping checkpoints.

Targeting mitochondrial stress—specifically preventing mtDNA release and Z-DNA formation—upstream of ZBP1, along with selective inflammasome suppression downstream (NLRP3–caspase-1–GSDMD), offers synergistic leverage to collapse PANoptotic escalation by removing its pyroptotic fuel and initiation triggers. Biomarker-guided sequencing, including early mitophagy/mtROS control followed by inflammasome/GSDMD inhibition, aligns with the temporal rise of pyroptosis (peak around day 7) and the conditions favoring PANoptosome assembly. This approach improves the odds of interrupting the shared execution machinery before mixed-death amplification ensues.

### 7.2 Ferroptosis and autophagy

Ferroptosis–autophagy crosstalk in SCI microglia is defined by autophagic pathways that both promote and restrain iron-catalyzed lipid peroxidation. NCOA4-dependent ferritinophagy increases labile iron, which fuels ferroptosis, while lysosome competence, governed by transcription factor EB (TFEB), and selective cargo control can oppose ferroptosis by maintaining organelle quality and limiting redox stress. Conversely, iron overload and lipid peroxidation feedback to provoke autophagy and lysosomal responses, creating phase- and context-dependent loops that can either escalate injury or enable repair when appropriately tuned.

HO-1 induction, coupled with NCOA4-dependent ferritinophagy, liberates catalytic iron from ferritin, driving glutathione depletion, GPX4 failure, and lipid peroxidation, which accelerates ferroptotic death in microglia and macrophages after SCI. Additionally, the lysosomal membrane protein GLMP acts as an intrinsic brake on ferroptosis. Loss of GLMP increases microglial iron deposition, ACSL4 and COX-2 expression, mitochondrial damage, and behavioral deficits, thereby linking lysosome integrity (an autophagy endpoint) to ferroptosis control.

Systemic and intracranial iron overload activates microglial NF-κB, provoking autophagy and proinflammatory cytokine release that contribute to neurovascular pathology and central pain. This demonstrates that ferroptotic upstream signals can drive autophagy programs in microglia. Restoring autophagic flux, such as through TFEB/lysosome competence or mitophagy, lowers mitochondrial ROS and removes damage-associated cargo. This reduces lipid peroxidation inputs that otherwise sustain ferroptosis. However, excessive or mistimed autophagy can be deleterious, underscoring the need for phase-specific tuning to prevent harmful outcomes.

Autophagy completion depends on lysosomal competence, which simultaneously governs ferritin turnover (ferritinophagy) and the GLMP brake on ferroptosis. This positions the lysosome as a decisive node where cargo routing can tip iron balance toward or away from ferroptosis. Additionally, mitophagy reduces mitochondrial ROS (mtROS) that feed lipid peroxidation. Ferroptotic microglia exhibit lipid peroxidation-induced mtROS that amplify inflammatory signaling, identifying mitochondrial redox as a shared lever connecting both pathways in SCI.

Pairing early ferroptosis inhibitors or iron chelation (e.g., Ferrostatin 1 or UAMC 3203) with autophagy restoration (e.g., TFEB activation or mitophagy support) can collapse iron–lipid peroxidation feedback while preventing autophagy stall and lysosomal stress.

Finally, integrating a standardized ferroptosis panel with autophagy flux markers, such as GPX4/GSH, 4-HNE/MDA, ACSL4, labile iron pools, and dynamic LC3 II/p62 with lysosomal metrics, will help clarify when autophagy protects from, *versus* precipitates, ferroptosis in phase-specific SCI windows.

### 7.3 Pyroptosis and autophagy

Intact autophagy restrains the NLRP3–caspase-1–GSDMD pyroptotic axis by degrading inflammasome components and lowering mitochondrial ROS through mitophagy, while inflammasome activity can feedback to suppress or mis-set autophagy levels. This creates a bidirectional and context-dependent relationship. Across various interventions, selectively re-engaging autophagic flux or mitophagy through pathways like AMPK/ULK1, TFEB/lysosome, and PINK1–Parkin consistently reduces ASC specks, caspase-1 activation, GSDMD cleavage, and IL-1β/IL-18 releases. Blocking autophagy, however, abolishes these anti-pyroptotic gains, confirming the mechanistic crosstalk between autophagy and pyroptosis.

In terms of core mechanisms, bidirectional coupling is evident as inhibiting NLRP3 with MCC950 reduces Beclin-1/LC3 levels and raises p62, dampening autophagy, while inducing autophagy *via* AMPK/ULK1 drives K48-ubiquitination and autophagic degradation of NLRP3. This shift promotes a reparative M2 phenotype in microglia and improves functional outcomes. Additionally, mitophagy acts as a brake on pyroptosis, with compounds like urolithin A and ginkgolide B activating PINK1–Parkin mitophagy to clear damaged mitochondria, reduce mtROS, and block ASC speck formation and caspase-1 activation. The protective effects of mitophagy are abolished by autophagy inhibitors like 3-MA, BafA1, or Parkin knockdown, proving mitophagy as a central anti-pyroptotic lever. Furthermore, lipophagy failure, due to CD36-mediated suppression of AMPK/TFEB, chokes autophagosome/lysosome biogenesis, while CD36 inhibition restores lipophagic flux, empties lipid droplets, and curbs microglial pyroptosis, linking lipid-clearance autophagy to inflammasome control.

Upstream regulators of this crosstalk include energy signaling, where CB2 agonism (JWH-133) enhances autophagy through AMPK/ULK1 and targets NLRP3 for autophagic degradation, while physical modalities like photobiomodulation and EA normalize autophagic markers and suppress the NLRP3–caspase-1–IL-1β cascade *in vivo*. Non-coding RNAs and zinc also play significant roles; miR-99b-3p boosts autophagic flux and suppresses NLRP3, attenuating caspase-1/GSDMD pyroptosis, while zinc restores autophagic flux, upregulates miR-374a-5p, and promotes autophagy-driven proteolysis of NLRP3. In both cases, autophagy inhibition reinstates inflammasome activity. The lysosome/TFEB axis is also critical, as trehalose activates TFEB to restore lysosome-dependent autophagic flux, dampening innate inflammatory pathways including NLRP3 signaling and improving recovery. This underscores lysosomal competence as a shared checkpoint for autophagy–pyroptosis crosstalk.

In execution readouts, autophagy engagement correlates with reduced inflammasome assembly (ASC specks), caspase-1 cleavage, GSDMD processing, and IL-1β/IL-18 efflux. Pharmacologic or genetic blockade of autophagy (e.g., 3-MA, BafA1, Parkin knock-down) reverses these effects and restores pyroptotic signaling in microglia. Conversely, direct inflammasome suppression can shift autophagy; MCC950 lowers autophagic markers *via* an mTOR-linked route, and rapamycin rescues both autophagy and phenotype, indicating feedback from NLRP3 into autophagy set-points.

The therapeutic implications of these findings suggest that restoring autophagy is key. Benefits track with re-establishing optimal flux rather than maximal induction, restraining pathological inflammasome activity without tipping into autophagy-dependent cell death, which can emerge with prolonged or excessive activation under severe injury. Rational sequencing of treatments—early mitophagy and TFEB/lysosome repair to lower mtROS and route NLRP3 for degradation, followed by sustained AMPK–mTOR tuning—can collapse the pyroptotic feed-forward loop and maintain an anti-inflammatory M2 microglial phenotype *in vivo*.

Notable exemplars of interventions include the CB2 agonist JWH-133, which drives AMPK/ULK1-driven autophagy and promotes M2 polarization with reduced neuroinflammation and better function. Urolithin A or ginkgolide B activate PINK1–Parkin mitophagy to lower mtROS and block ASC/caspase-1/GSDMD, with autophagy blockade abrogating all benefits. Zinc modulation offers dual action by restoring autophagic flux and promoting autophagic proteolysis of NLRP3; autophagy inhibitors negate protection, confirming the mechanism. Non-pharmacologic control through photobiomodulation and EA has also been shown to restore autophagy markers and suppress NLRP3–caspase-1–IL-1β signaling, demonstrating that physical modalities can regulate the coupling between autophagy and pyroptosis.

Despite these promising findings, gaps remain in the current research. Most data come from rodent models with bulk LC3/p62 readouts, and true *in vivo* flux, cell-type specificity, and long-term safety remain to be defined for precise autophagy tuning against pyroptosis. Further research is needed to systematically map the intersections between autophagy modes (mitophagy, lipophagy) and distinct inflammasomes (NLRP3 vs. AIM2) to identify master switches that collapse pyroptosis without off-target stress or autophagic cell death.

### 7.4 Autophagy and necroptosis

Disruption of autophagic flux, through lysosomal dysfunction and mTOR hyperactivation, leads to the accumulation of RIPK1 and RIPK3, sensitizing cells to necroptosis. Restoring autophagy through AMPK–mTOR pathways reduces necroptotic priming and inflammation *in vivo*. Intercellular signaling further links the two processes, as necroptotic NSCs export TSC2 mRNA that restrains mTOR signaling, favoring autophagy-mediated clearance and suggesting a negative-feedback loop that modulates chronic inflammation.

Key mechanisms further elaborate this connection. Disruption of autophagy primes necroptosis after SCI. Lysosomal dysfunction and mTOR hyperactivation stall autophagic flux in microglia, causing RIPK1 and RIPK3 accumulation, which sensitizes the cells to necroptosis. This directly ties impaired autophagy to the necroptotic execution machinery in injured tissue and microglial cultures. The intersection with endoplasmic reticulum stress is also notable, as endoplasmic reticulum stress colocalizes with RIPK3/MLKL during necroptosis in post-traumatic microglia. The collapse of autophagic flux activates the inflammasome and primes necroptosis, highlighting the convergence of stress, autophagy, and RIPK signaling. Restoring autophagy reduces necroptotic pressure, with pharmacological autophagy induction through rapamycin or resveratrol reactivating AMPK/mTOR–Beclin-1 signaling, suppressing microglial pro-inflammatory activation, and positioning autophagy as a counterweight to the necroptosis-permissive inflammatory milieu following flux failure.

Intercellular feedback mechanisms are also significant. Necroptotic ependymal-derived NSCs export exosomes carrying TSC2 mRNA. The uptake of these exosomes restrains mTOR signaling in peri-lesional cells, promoting autophagy-mediated debris clearance and implying that necroptosis can secondarily stimulate autophagy as an adaptive brake on chronic inflammation. The mTOR axis plays a central role in this crosstalk, as mTOR hyperactivation impairs autophagic flux and primes necroptosis, while restraining mTOR *via* AMPK activation or TSC2 delivery restores autophagy and reduces necroptotic pressure in the lesion microenvironment. The accumulation of RIPK1 and RIPK3 during autophagy disruption mechanistically links these necroptosis effectors to the autophagic status. Integrative remarks emphasize that ER-stress–RIPK3/MLKL crosstalk is a key intersection with autophagy failure, contributing to necroptosis initiation.

Therapeutically, re-engaging autophagy through AMPK activation or mTOR inhibition (like rapamycin or resveratrol) restores Beclin-1/LC3 dynamics and reduces pro-inflammatory activation, which accompanies necroptosis priming. This suggests that timed autophagy induction can blunt necroptotic cascades. The use of exosomal TSC2 from necroptosis-conditioned NSCs to deliver TSC2 has been suggested as a strategy to restrain mTOR and enhance autophagy-mediated clearance, rebalancing autophagy–necroptosis signaling. A programmatic roadmap calls for the conditional knock-in/knock-out of RIPK3 and autophagy genes in microglia *versus* infiltrating macrophages to resolve initiators *versus* amplifiers and define stage-specific windows for autophagy-guided necroptosis control.

The directionality and timing of these processes are crucial. The collapse of autophagic flux primes necroptosis, indicating that the timing of flux failure during acute and sub-acute phases is a key determinant of RIPK–MLKL pathway engagement in microglia and neighboring compartments. Conversely, necroptotic NSCs initiate a compensatory TSC2–mTOR–autophagy axis through exosome transfer, which is predicted to enhance debris clearance. This suggests that necroptosis-triggered paracrine cues can recalibrate autophagy to restrain chronic inflammation, creating a feedback loop between the two processes.

There are several evidence gaps in understanding these mechanisms. Most data come from rodent models with limited *in vivo* resolution of autophagic flux and lack microglia-specific genetics across chronic timelines. Literature recommends microglia-targeted manipulation of RIPK3 and autophagy genes to clarify causal directionality and therapeutic windows. Additionally, integrating endoplasmic reticulum stress metrics with dynamic autophagy and necroptosis readouts at single-cell resolution is necessary to determine when restoring autophagy suppresses or fails to affect RIPK–MLKL execution in distinct SCI phases.

### 7.5 Necroptosis and ferroptosis

Ferroptosis and necroptosis intersect in SCI primarily through an iron–inflammation and organelle-stress axis. Iron-driven lipid peroxidation fuels a proinflammatory TNF-α loop that conditions tissues for RIPK1/RIPK3/MLKL execution, while mitochondrial and endoplasmic reticulum stress from ferroptotic signaling converge on the necroptotic machinery through RIPK3/MLKL at sites of ER stress colocalization. Ferroptotic microglia amplify oxidative and cytokine pressure, such as IL-1β, IL-6, IL-23, and TNF-α, which propagates necroptosis within microglia and to the vascular niche. Conversely, dampening iron-lipid damage or stress signaling can blunt both death programs *in vivo*.

Iron and TNF-α play critical roles in the mechanistic overlap between ferroptosis and necroptosis. Iron overload and lipid peroxidation in microglia elevate TNF-α levels, reinforcing an M1 state that sustains oxidative injury. This TNF-α pressure, in turn, drives endothelial RIPK1/RIPK3/MLKL activation and blood–spinal-cord-barrier breakdown, linking ferroptotic inflammation to necroptotic execution across compartments. Additionally, ferroptotic microglia generate mtROS and engage PERK-linked stress pathways, with endoplasmic reticulum stress colocalizing with RIPK3/MLKL during necroptosis. This intersection is mitigated by the chemical chaperone 4-phenylbutyrate, indicating shared stress circuitry between ferroptosis and necroptosis.

The shared drivers of ferroptosis and necroptosis include oxidative burden and cytokine release. Lipid peroxidation, such as that involving 4-HNE, and ROS amplify NF-κB signaling, which in turn escalates ferroptosis in microglia and promotes necroptotic susceptibility in adjacent cells *via* inflammatory priming. Iron-induced oxidative stress locks microglia into an iNOS^+^/CD86^+^ M1 phenotype, propagating ferroptosis and contributing to the TNF-α milieu that permits RIPK-MLKL necroptosis when caspase-8 restraints are compromised.

The directionality of these processes’ links microglial activity to endothelial injury. Microglial TNF-α initiates endothelial necroptosis, coupling ferroptosis-driven inflammation in microglia to necroptotic vascular injury and secondary leukocyte infiltration. Intracellular stress tipping occurs as ferroptosis-linked organelle stress and ROS push microglia toward RIPK3/MLKL activation under ER-stress conditions. This integrated analysis highlights the endoplasmic reticulum stress–RIPK3/MLKL intersection as a seed for necroptosis during oxidative injury.

Therapeutically, dual-axis targeting is a promising strategy. Combining ferroptosis control, such as through iron chelation, GPX4/GSH restoration with ferrostatin-1 or UAMC-3203, or antioxidant nanoplatforms, with necroptosis interception, such as reducing TNF-α *via* VNS or dampening endoplasmic reticulum stress with 4-phenylbutyrate, is a rational approach to break both the lipid-ROS and RIPK-MLKL arms. Multifunctional biomaterials that chelate iron, quench ROS, and restore GPX4 not only restrain ferroptosis but also lower upstream inflammatory and stress signals that feed necroptosis, aligning with the need for combinatorial control of iron-lipid and RIPK-MLKL pathways.

Despite these insights, several evidence gaps remain. Most data come from rodent models and infer crosstalk from shared mediators. Single-cell genetics that jointly manipulate GPX4/iron handling and RIPK3/MLKL in microglia *versus* infiltrating macrophages are needed to resolve causality and stage-specific windows. Additionally, standardized readouts that track both ferroptosis (GPX4/ACSL4/4-HNE) and necroptosis (RIPK1/RIPK3/p-MLKL) in the same experiments will clarify whether suppressing ferroptotic stress or endoplasmic reticulum stress is sufficient to curb necroptotic spread across neural and vascular compartments.

### 7.6 Combinatorial therapeutic strategies targeting intersecting death pathways

Therapeutic strategies targeting the intersection of programmed cell death pathways in microglia highlight several promising combinatorial approaches (Table 2). One emerging concept is the pairing of autophagy restorers, particularly through the AMPK/mTOR axis, with ferroptosis blockers. Agents such as metformin, rapamycin, or trehalose can rejuvenate autophagy and mitophagy, thereby clearing damaged mitochondria and lipid droplets to reduce mitochondrial ROS and inflammatory priming. When combined with ferroptosis inhibitors such as ferrostatin-1, UAMC-3203, or Idebenone–FSP1 axis modulators, these regimens restore GPX4/GSH activity and suppress iron-driven lipid peroxidation. Together, these interventions interrupt the iron–TNF–ferroptosis feed-forward loop in microglia, supporting M2 repolarization and protecting neurons.

**TABLE 2:** Summary of combinatorial therapeutic strategies targeting microglial programmed cell death after SCI.

Strategy	Possible pairs	Rationale
Autophagy Restorers (AMPK/mTOR axis) + Ferroptosis Blockers	Metformin/Rapamycin/Trehalose + Ferrostatin-1/UAMC-3203/Se-nanoplatforms/Idebenone–FSP1	Restores autophagy/mitophagy and suppresses lipid peroxidation; interrupts iron–TNF–ferroptosis loop, supports M2 repolarization and neuronal sparing.
TFEB-Driven Lipophagy Boost + CD36 Inhibition	Trehalose (TFEB activator) + Sulfo-N-succinimidyl-oleate (CD36 blocker)	Enhances lysosome–autophagy *via* TFEB and removes CD36 brake; accelerates lipid clearance, reduces pyroptosis, improves recovery.
Temporal Sequencing: Early Ferroptosis Blockade → Later Pyroptosis Brake	UAMC-3203 or Ferrostatin-1 (acute) → MCC950 (NLRP3) or exosomal miR-22 (anti-GSDMD) ∼day 7	Matches interventions to dominant windows: early ferroptosis inhibition for redox/membrane protection, later pyroptosis inhibition to limit cytokine release.
AMPK/mTOR Autophagy Modulation + Nrf2 Antioxidant Activation	Metformin or Rapamycin + UAMC-3203 (Nrf2/HO-1)/Cynarin/Gallic Acid	Reduces mtROS and inflammatory signaling *via* autophagy; Nrf2 activation restores GPX4/GSH, suppressing lipid peroxidation and NLRP3 priming.
Zinc-Centric Mitophagy Rescue + Autophagy/Mitophagy Enhancers	Zinc supplementation + Metformin/Rapamycin/Celastrol–Metformin–Everolimus nano-delivery	Zinc reinstates mitophagy and VEGF-A survival signaling, breaks iron–TNF–ferroptosis loop; combined with AMPK/mTOR enhancers rebuilds mitochondrial QC.
PANoptosis-Aware Pairing: Ferroptosis Blockade + Melatonin	Ferrostatin-1 or UAMC-3203 + Melatonin (MT1/MT2-mediated anti-pyroptosis)	Addresses concurrent PANoptosis pathways; ferroptosis blockade reduces lipid peroxidation, melatonin restrains pyroptosis *via* NLRP3/caspase-1/GSDMD.
Microtubule-Guided Regeneration + Anti-Ferroptotic Support	Paclitaxel–Idebenone nanomicelles ± short acute ferroptosis blocker	Nanomicelles support axon regrowth and FSP1 ferroptosis suppression; short acute ferroptosis blockade enhances early protection.
Chemokine-Autophagy Rebalance + Ferroptosis Inhibition	BX471 (CCR1 antagonist) + Ferrostatin-1/UAMC-3203	CCR1 antagonism restores autophagy flux and reduces TNF-α/IL-1β; ferroptosis inhibition prevents lipid peroxidation sustaining inflammatory states.

A second strategy builds on TFEB-driven lipophagy induction in tandem with CD36 inhibition. Lipid droplet accumulation is increasingly recognized as a barrier to effective autophagy and a driver of pyroptotic tone in microglia. Activating TFEB with trehalose enhances lysosome–autophagy capacity, while blockade of CD36 using sulfo-N-succinimidyl-oleate removes a major brake on AMPK/TFEB signaling. This dual approach accelerates lipid clearance, dampens pyroptotic signaling, and facilitates recovery, with evidence suggesting additive benefits when combined.

Timing also appears crucial, as ferroptosis and pyroptosis dominate distinct phases of injury progression. An early course of ferroptosis inhibition with UAMC-3203 or ferrostatin-1 preserves membrane integrity and redox balance during the acute phase, while delayed introduction of pyroptosis inhibitors such as MCC950 (targeting NLRP3) or exosomal miR-22 (suppressing GSDMD) around day seven may better align with pyroptotic peaks observed *in vivo*. Such temporal sequencing tailor intervention to the evolving death programs of microglia.

In parallel, coupling AMPK/mTOR-mediated autophagy restoration with Nrf2-driven antioxidant activation offers another two-pronged approach. Metformin or rapamycin enhance mitochondrial clearance, reducing sources of oxidative stress, while agents such as UAMC-3203, cynarin, or gallic acid boost the Nrf2/HO-1 axis to replenish GPX4/GSH levels and suppress inflammasome priming. This combined modulation addresses both the origin of oxidative injury and its downstream inflammatory consequences.

Zinc supplementation also represents a key adjunct, as it reinstates mitophagy *via* STAT3/FoxO3a/SOD2 pathways, supports VEGF-A–linked microglial and endothelial survival, and disrupts the iron–TNF–ferroptosis cycle. When paired with autophagy enhancers such as metformin, rapamycin, or nanoplatform-delivered drug combinations, zinc may synergistically rebuild mitochondrial quality control and attenuate inflammatory death cascades.

Recognizing the overlapping nature of programmed cell death, PANoptosis-aware strategies pair ferroptosis inhibition with melatonin. While ferrostatin-1 or UAMC-3203 blunt lipid peroxidation, melatonin constrains microglial pyroptosis through MT1/MT2-mediated suppression of NLRP3, caspase-1, and GSDMD. This combined approach addresses the multi-arm PANoptotic signaling observed in ischemia-reperfusion and spinal cord injury models.

Other approaches directly combine regenerative support with ferroptosis blockade. Paclitaxel–idebenone nanomicelles not only stabilize axonal microtubules but also restore FSP1-dependent ferroptosis suppression. Adding a short course of a GPX4-pathway ferroptosis inhibitor during the acute phase could provide additional early membrane protection while the nanomicelles sustain longer-term pro-regenerative and anti-ferroptotic effects.

Finally, rebalancing chemokine signaling with autophagy regulation represents a further avenue. CCR1 antagonism with BX471 restores autophagic flux by normalizing Beclin-1/LC3-II and reducing p62 accumulation, while simultaneously lowering TNF-α and IL-1β production. Combining this with ferroptosis inhibition directly counters lipid peroxidation and the inflammatory microglial states it promotes.

Together, these integrative strategies illustrate how carefully designed combinations—spanning autophagy restoration, ferroptosis inhibition, pyroptosis restraint, antioxidant activation, and regenerative support—can disrupt self-amplifying microglial death programs and improve neuronal outcomes.

## 8 Conclusions and future remarks

SCI unleashes a spectrum of programmed cell death axes in microglial cells that extends far beyond classical apoptosis. Ferroptosis, pyroptosis, necroptosis and the emerging umbrella of PANoptosis all operate in parallel with autophagy and autophagy‐derived processes such as mitophagy and lipophagy. These pathways are not siloed: autophagic flux restrains NLRP3-driven pyroptosis, while its collapse licenses inflammasome activation and primes necroptosis; iron-induced lipid peroxidation feeds a TNF-α loop that locks microglia into an M1, ferroptosis-propagating state; and endoplasmic reticulum stress signalling intersects RIPK3/MLKL to seed necroptosis that radiates to endothelial and stem-cell compartments. The result is a self-reinforcing matrix of oxidative damage, ion dys-homeostasis and cytokine storm that drives secondary degeneration, chronic neuroinflammation and remote metabolic sequelae.

Despite this complexity, therapeutic studies converge on a handful of molecular choke-points. Small molecules (rapamycin, metformin, quercetin), nutraceuticals (fisetin, salidroside), trace-element supplementation (zinc) and neuromodulatory procedures (HVPRF, VNS, photobiomodulation) all restore AMPK- or mTOR-regulated autophagy and shift microglia toward a reparative M2 phenotype. Nanoplatforms add simultaneous iron chelation, ROS quenching and GPX4 rescue to blunt ferroptosis and re-program innate immunity. Gene, miRNA and exosome approaches fine-tune single nodes within the NLRP3–caspase-1–GSDMD or RIPK3–MLKL axes with remarkable precision and behavioral benefit.

Yet several translational gaps persist. Comparative studies rarely extend beyond young male rodents; chronic phases and comorbidity settings are understudied. Ferroptosis work suffers from heterogeneous biomarker panels that hamper head-to-head evaluation, and ncRNA datasets contain cell-type or timing contradictions (e.g., circ0000381) that remain unresolved. Off-target effects of systemic autophagy activation, long-term biodistribution of metal or magnetite-based carriers, and the safety of repeated neuromodulation all demand clarification.

### 8.1 Future directions

Single-cell and spatial transcriptomics, proteomics and lipidomics across acute, sub-acute and chronic windows will map how microglial programmed cell death signatures evolve and interact with neurons, astrocytes, endothelial cells and peripheral organs (e.g., Leydig-cell lipophagy blockade).

Conditional knock-in/knock-out models for RIPK3, GSDMD, GPX4, ZBP1 and autophagy genes in microglia *versus* infiltrating macrophages will disentangle initiators from amplifiers and reveal stage-specific therapeutic windows.

Rational combinations that pair, for example, an early ferroptosis chelator with mid-phase autophagy modulators and late anti-pyroptotic miRNAs should be benchmarked against monotherapies in large-animal models.

Streamlined carriers that hit the dominant ferroptotic or pyroptotic lever without unnecessary complexity will ease regulatory hurdles while chronic retention and systemic redistribution are assessed in primate studies.

Post-mortem and biopsy material, combined with blood or CSF “programmed cell death fingerprints”, will validate rodent findings and provide pharmacodynamic read-outs for first-in-human trials.

Standardized manufacturing, potency assays and long-term oncogenicity testing must accompany the rapid progress of viral, CRISPR and EV-based interventions.

Hormonal milieu, metabolic status and immune senescence all may modulate microglial death phenotypes; future trials should incorporate these variables into design and analysis.

Aligning HVPRF, Vagus-nerve or optical stimulation paradigms with physical rehabilitation could synchronize circuit plasticity with microglial re-education for additive functional gains.

Direct demonstration of microglial PANoptosome assembly in SCI and systematic mapping of lipophagy–ferroptosis crosstalk may reveal master switches capable of collapsing multiple injurious pathways at once.

By integrating these lines, the field can progress from descriptive catalogues of microglial death to precision, phase-specific interventions that not only spare tissue but also orchestrate true regeneration.

### 8.2 Current status of clinical translation

Although the majority of experimental studies on programmed cell death in SCI have been conducted in rodent models, a number of clinically available agents have been reported in preclinical research to exert their effects, at least in part, through the regulation of cell death pathways. This section highlights the association between drugs already in clinical use and their potential mechanisms of action in SCI, focusing particularly on their modulation of autophagy, apoptosis, and pyroptosis.

Among currently approved therapies, methylprednisolone remains the only glucocorticoid widely administered in the acute management of SCI. Beyond its classical anti-inflammatory effects, recent evidence indicates that methylprednisolone promotes a *Beclin-1–dependent autophagic program* in over-activated microglia. This process is mediated through upregulation of the zinc importer ZIP8 and expansion of the labile zinc pool, which in turn suppresses NF-κB activation. By inducing protective autophagic death in pathological microglia, methylprednisolone reduces excessive neuroinflammation and preserves neuronal integrity. Importantly, zinc chelation abolishes these protective effects, underscoring the requirement of zinc in this pathway.

Metformin, a first-line therapy for type 2 diabetes, has also been investigated in SCI models, where it activates AMPK and inhibits mTOR signaling. This shift restores autophagosome–lysosome fusion in microglia, thereby re-establishing autophagic flux. The result is a repolarization of microglia from a pro-inflammatory M1 phenotype toward an anti-inflammatory M2 phenotype, enhanced clearance of myelin debris, and improved white matter preservation. The neuroprotective actions of metformin are abolished when autophagy is pharmacologically blocked, demonstrating that its beneficial effects are mediated directly through autophagy-dependent mechanisms of programmed cell death.

Rapamycin (sirolimus) and its analog everolimus, both clinically employed as immunosuppressants, have also shown promise in experimental SCI. Rapamycin suppresses mTORC1 activity, increases Beclin-1 and LC3-II expression, and restores autophagic flux in the injured spinal cord. These changes correlate with reduced infiltration of inflammatory microglia/macrophages, lower TNF-α release, and enhanced neuronal survival. Similarly, everolimus, when incorporated into a nanoplatform with metformin and celastrol, synergistically promotes autophagy and mitophagy, attenuates inflammatory cytokine release, and fosters functional recovery. Taken together, these findings suggest that clinically available mTOR inhibitors can beneficially modulate autophagy-linked cell death pathways in SCI.

Zinc, an essential micronutrient with established clinical use, has been implicated in the regulation of both autophagy and pyroptosis following SCI. Systemic zinc administration reactivates mitophagy through a STAT3/FoxO3a/SOD2 signaling axis, reducing mitochondrial ROS production, improving ATP generation, and supporting functional recovery. Zinc has also been reported to suppress inflammasome activity by restoring autophagic flux, leading to enhanced degradation of NLRP3 and reduced pyroptosis. These data highlight zinc as a clinically available modulator of PCD in the injured spinal cord, consistent with its role as a co-factor in methylprednisolone-mediated autophagic signaling.

While these findings illustrate encouraging intersections between clinically used drugs and programmed cell death pathways, it is important to recognize that the evidence is largely restricted to rodent studies. No large-scale clinical trials have yet validated these mechanisms or confirmed long-term efficacy in human SCI patients. Furthermore, careful attention to dosing and timing will be critical, since excessive or prolonged autophagy can itself promote cell death under conditions of severe energy stress. Future clinical translation will therefore require rigorously designed trials to determine the safety, efficacy, and therapeutic window of these agents in modulating PCD for SCI treatment.
